# RNA Polymerase Pausing during Initial Transcription

**DOI:** 10.1016/j.molcel.2016.08.011

**Published:** 2016-09-15

**Authors:** Diego Duchi, David L.V. Bauer, Laurent Fernandez, Geraint Evans, Nicole Robb, Ling Chin Hwang, Kristofer Gryte, Alexandra Tomescu, Pawel Zawadzki, Zakia Morichaud, Konstantin Brodolin, Achillefs N. Kapanidis

**Affiliations:** 1 Biological Physics Research Group, Clarendon Laboratory, Department of Physics, University of Oxford, Oxford OX1 3PU, UK; 2 CNRS FRE 3689, Centre d’études d’agents Pathogénes et Biotechnologies pour la Santé (CPBS), 1919 route de Mende, 34293 Montpellier, France

**Keywords:** RNA polymerase, initial transcription, DNA scrunching, transcriptional pausing, single-molecule FRET, promoter escape

## Abstract

In bacteria, RNA polymerase (RNAP) initiates transcription by synthesizing short transcripts that are either released or extended to allow RNAP to escape from the promoter. The mechanism of initial transcription is unclear due to the presence of transient intermediates and molecular heterogeneity. Here, we studied initial transcription on a *lac* promoter using single-molecule fluorescence observations of DNA scrunching on immobilized transcription complexes. Our work revealed a long pause (“initiation pause,” ∼20 s) after synthesis of a 6-mer RNA; such pauses can serve as regulatory checkpoints. Region sigma 3.2, which contains a loop blocking the RNA exit channel, was a major pausing determinant. We also obtained evidence for RNA backtracking during abortive initial transcription and for additional pausing prior to escape. We summarized our work in a model for initial transcription, in which pausing is controlled by a complex set of determinants that modulate the transition from a 6- to a 7-nt RNA.

## Highlights

• *E. coli* RNA polymerase pauses during initial transcription at *lac* promoters
• Initiation pausing lasts for ∼20 s and occurs at the transition from 6- to 7-nt RNA
• Region 3.2 of σ^70^ is the main protein element controlling pausing
• Pausing is likely to be controlled further by a complex set of determinants

## Graphical Abstract

**Figure:**
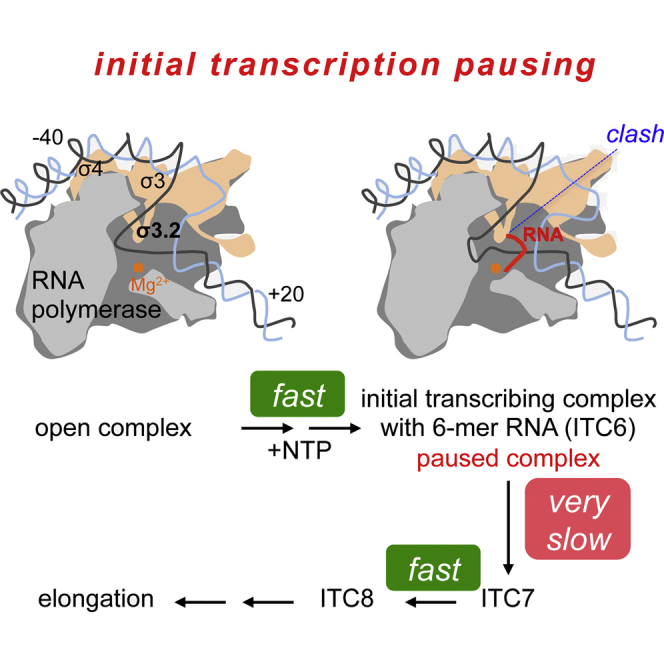

## Teaser

Initial transcription involves synthesis of short RNAs that are either released or extended to allow promoter escape; such mechanisms are unclear due to heterogeneity. Duchi et al. used single-molecule fluorescence to visualize transcription in real time and discovered a long pause after synthesis of a 6-mer RNA.

## Introduction

Transcription initiation is the most highly regulated step in gene expression. In bacteria, RNA polymerase (RNAP) binds to promoter DNA and unwinds ∼14 bp around the transcription start site to form a transcription bubble, with the unwound template (T) strand moving into the RNAP active center cleft. This conformational change leads to the formation of the RNAP-promoter open complex, RP_o_ (Murakami and Darst, 2003, Saecker et al., 2011), which then engages in de novo RNA synthesis via productive or abortive pathways (Carpousis and Gralla, 1980, Hsu, 2002). In the productive pathway, RNAP synthesizes RNA within an RNAP-promoter initial transcribing complex (ITC); when the nascent RNA becomes 9- to 11-nt long, RNAP escapes from the promoter and enters elongation (Mukhopadhyay et al., 2001, Murakami and Darst, 2003). In the abortive pathway (also known as abortive initiation), RNAP synthesizes short RNAs, but does not escape from the promoter; instead, RNAP releases short RNAs, reverts back to RP_o_, and re-initiates RNA synthesis (Carpousis and Gralla, 1980, Gralla et al., 1980). The balance between productive and abortive pathways depends on the promoter and initial transcribed sequences (Hsu, 2009).

Despite this progress, which has been aided by structures of ITCs (Basu et al., 2014, Zuo and Steitz, 2015), our understanding of initial transcription is limited, in part due to the heterogeneity and dynamics of the complexes involved (Hsu, 2002, Hsu, 2009, Kubori and Shimamoto, 1996). Such issues are addressable by single-molecule studies, which can also examine reactions in real time without synchronization. In early work, we used single-molecule Förster resonance energy transfer (smFRET) confocal microscopy (Kapanidis et al., 2004, Kapanidis et al., 2005a) to monitor multiple distances within diffusing transcription complexes and showed that initial transcription proceeds via a DNA-scrunching mechanism (Kapanidis et al., 2006), during which RNAP unwinds and pulls downstream DNA into its active site cleft. DNA nanomanipulation work also showed that scrunching occurs in initial transcription and is obligatory for escape (Revyakin et al., 2006).

However, the confocal smFRET study offered only short (∼1 ms) structural snapshots of transcription complexes. An early smFRET work on immobilized complexes (Margeat et al., 2006) was also limited by low temporal resolution, short observations, and photophysical fluctuations. In contrast, the DNA nanomanipulation work offered long observations, but did not identify kinetically stable intermediates. As a result, the mechanism, kinetics, and regulation of initial transcription have remained unclear. There is also a need to evaluate the role of σ^70^ region 3.2 (σ3.2) in initial transcription, since it is a major determinant of abortive initiation (Murakami et al., 2002).

**Figure 1:**
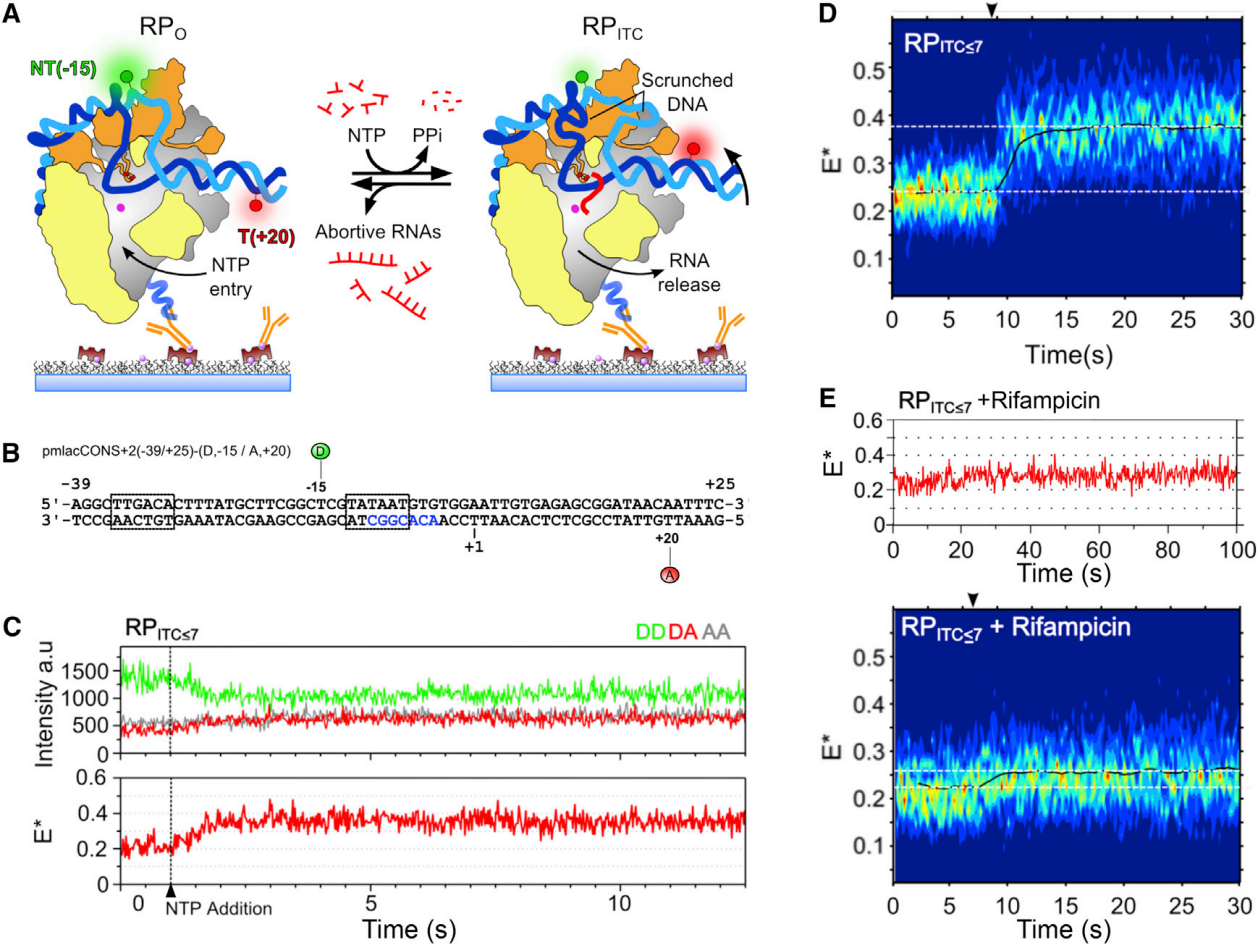
A Single-Molecule FRET Assay for Real-Time Initial Transcription (A) Schematic of assay. Left, RP_o_; right, initial transcribing complex (ITC). Donor is in green; acceptor in red; σ^70^ in orange; RNAP in gray, except for the β subunit (omitted for clarity) and regions protruding from the cut-away plane (in yellow); template strand in blue; non-template strand in teal; nascent RNA in red; and RNAP active site in pink. The penta-His antibody anchors RP_o_ to the surface. The initial FRET efficiency is low; upon NTP addition, scrunching moves the acceptor closer to the donor, increasing FRET efficiency. (B) *lac*CONS DNA fragment for FRET assay; the −10/−4 pre-melted region is in blue. (C) Time trace showing an increase to E^∗^∼0.37 upon adding 80 μM UTP and GTP to form RP_ITC≤7_. The NTP addition point is marked with a dashed line. Frame time: 20 ms. DD trace (green trace, top), donor emission upon donor excitation; DA trace (red trace, top), acceptor emission upon donor excitation; AA trace (gray trace, top), acceptor emission upon acceptor excitation. DD and DA are used for calculating apparent FRET efficiency E^∗^. (D) Transcription heatmaps (n = 45) showing activity upon NTP addition to form RP_ITC≤7_. NTP addition is marked by an arrowhead. Blue to red colors represent an increasing number of events. Black line, time trace of average E^∗^ of all traces; white dotted lines, E^∗^ for RP_o_ baseline (at E^∗^∼0.24) and RP_ITC_ plateau (at E^∗^∼0.37). Frame time: 200 ms. (E) Time trace (top) and transcription heatmap (bottom, n = 37) for RP_ITC≤7_ in the presence of rifampicin. See also Figure S1.

Here, we use an optimized smFRET strategy to monitor de novo RNA synthesis in real time by monitoring DNA scrunching, which occurs concomitantly with each nucleotide incorporation in initial transcription (Figure 1A). Surprisingly, we observe highly stable scrunched states and extensive pausing during initial transcription, with region σ3.2 being a major pausing determinant. We also obtained evidence for RNA backtracking during abortive initial transcription, and for additional pausing prior to escape. Our results were summarized in a model for initial transcription, in which pausing is controlled by a complex set of determinants that modulate the transition from a 6- to a 7-nt RNA.

## Results

### Real-Time Initial Transcription by Single RNAP Molecules

To study initial transcription in real time, we used smFRET to monitor DNA conformational changes within surface-immobilized transcription complexes. We used DNAs based on a derivative of *lac* promoter (*lac*CONS), a promoter rate-limited in initial transcription (Carpousis and Gralla, 1980, Gralla et al., 1980). We monitored FRET between fluorophores flanking the transcription bubble (Kapanidis et al., 2006, Margeat et al., 2006, Robb et al., 2013); the donor was placed in the −10/−35 spacer DNA (at position −15 of the non-template DNA) and the acceptor on the DNA downstream of the bubble (at position +20 of the template DNA; Figure 1A). The initial FRET efficiency for this pair in RP_o_ was expected to be low: as RNAP synthesizes short RNAs (2- to 7-mer), the downstream DNA flanking the acceptor should rotate and approach the donor, leading to a FRET increase (Figure 1A; for the expected donor-acceptor distances and FRET efficiencies, see Figure S1A, available online). To maximize the yield of active immobilized complexes, we used a pre-melted version of *lac* DNA (pmDNA; Figure 1B); the FRET pair on the DNA did not affect either the *lac* abortive profile or the ability of RNAP to escape (Figure S1B).

To measure the FRET efficiency in RP_o_ complexes, we anchored them to a polyethylene glycol (PEG)-coated surface and imaged them via total internal reflection fluorescence (TIRF) microscopy (Figure 1A). Immobilized RP_o_ in the presence of dinucleotide ApA (RP_ITC2_) formed the same stable FRET state as DNA alone (FRET efficiency [E^∗^]∼0.22; Figure S1C, top and middle panels) and did not reach higher FRET states (Figure S1D).

To observe initial transcription in real time, we provided immobilized RP_o_ complexes with subsets of nucleotides, trapping RNAP in iterative abortive synthesis and preventing promoter escape (Carpousis and Gralla, 1980, Kapanidis et al., 2006). Specifically, we added ApA, UTP, and GTP to form complexes limited to synthesis of RNA of up to 7 nt in length (RP_ITC≤7_; with the longest RNA being 5′-AAUUGUG-3′). Addition of 80 μM UTP and GTP (at ∼1 s; Figure 1C) indeed led to a gradual decrease in donor fluorescence and an anticorrelated increase in acceptor fluorescence (DD and DA traces; Figure 1C, top); these signals corresponded to a gradual FRET increase from the RP_o_ state (E^∗^∼0.2, initial segment of FRET trace; Figure 1C) to a higher FRET state (E^∗^∼0.37). After the initial increase (completed in ∼1 s), the FRET signal was stable, indicating that the E^∗^∼0.37 state is stable for >10 s.

To study all active complexes on a single field of view (n ∼ 50), we superimposed their FRET traces on a “transcription heat map” (Figure 1D). The map showed that the large majority of molecules display the same behavior of gradual increase (in 1–2 s) from RP_o_ to a higher FRET state (E^∗^ = 0.37 ± 0.01, mean ± SEM), which was occupied for >20 s. To test whether the increase was due to transcription, we performed controls wherein we added UTP and GTP to immobilized RP_ITC2_ in the presence of rifampicin, an inhibitor that blocks synthesis of RNA of >3 nt in length (Campbell et al., 2001, McClure and Cech, 1978). Our results showed only a small change (∼0.04) in the presence of rifampicin upon UTP/GTP addition (Figure 1E), likely due to the RNAP being able to extend ApA to a 3-nt RNA.

### RNAP Pauses after Synthesizing a 6-nt RNA

**Figure 2:**
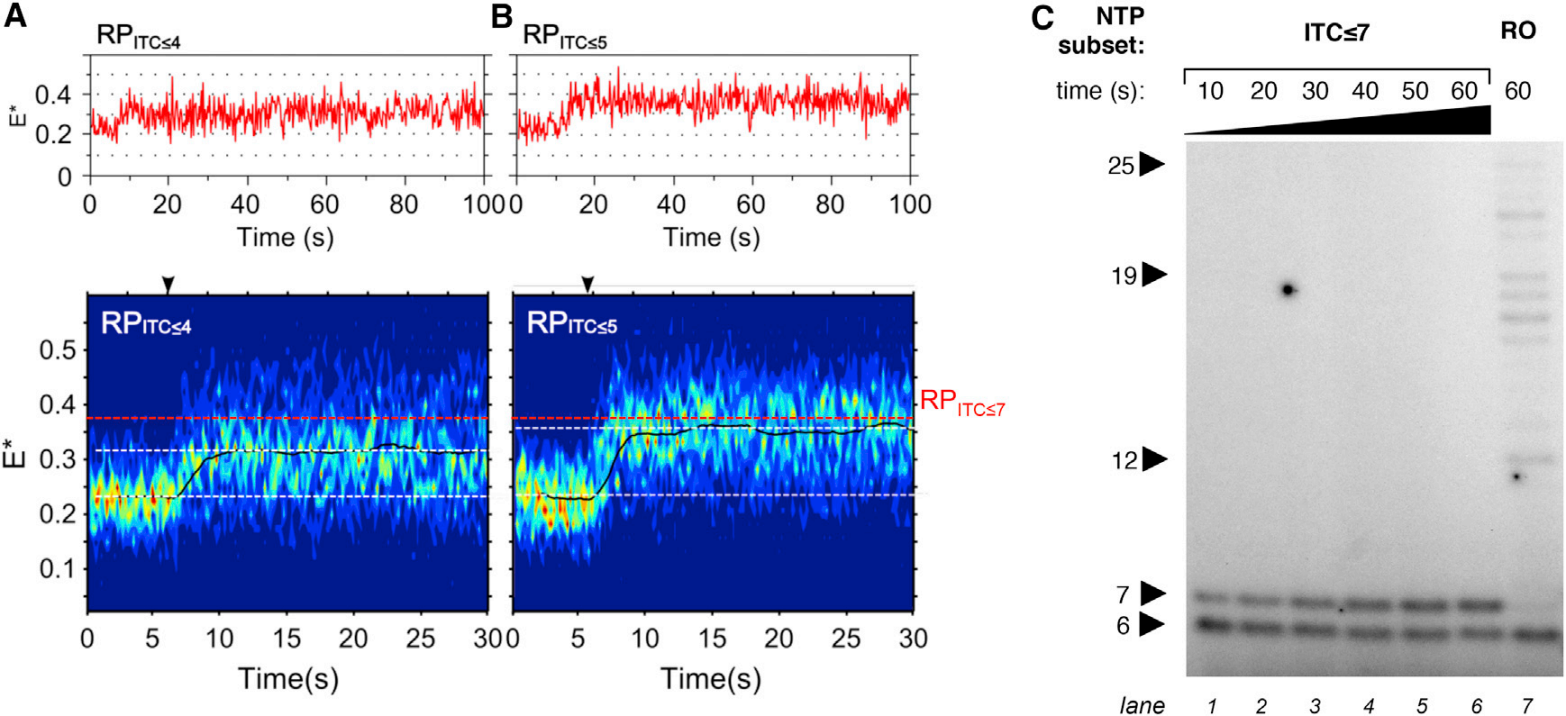
A Pause during RNA Extension from 6 to 7 nt in Length (A and B) FRET time traces (top) and heatmaps (bottom) for all active RP_ITC≤4_ (n = 45; A) and RP_ITC≤5_ (n = 53; B) complexes. Style as in Figure 1. The dotted red line at E^∗^∼0.37 marks the high-FRET plateau for RP_ITC≤7_ (Figure 1D). (C) Transcription activity for RP_ITC≤7_ and run-off products on *lac*CONS. Lanes 1–6 follow RNAs made under RP_ITC≤7_ conditions (RP_o_ + 500 μM ApA, 80 μM UTP, and 80 μM GTP) over 60 s. Lane 7 represents the run-off reaction (RP_o_ + 500 μM ApA, and 80 μM of all NTPs). The RNA length was assigned by comparison with length standards with sequences identical to the short RNAs produced on *lac*CONS; see Figure S2A. The gel shows no accumulation of RNAs shorter than 6 nt under our conditions; we note that 3- to 4-mers are also produced (see Figure 5F), but are not recovered well by the precipitation step prior to gel loading. See also Figure S2.

To monitor scrunching in different ITCs, we followed FRET during the first few nucleotide additions: we formed RP_ITC≤4_ and RP_ITC≤5_ complexes, generated their heatmaps, and compared them to RP_ITC≤7_ with regards to the magnitude of FRET increase and the stability of the highest FRET state (Figure 2A). For RP_ITC≤4_, a plateau at E^∗^∼0.32 was reached in ∼2 s after NTP addition (Figure 2A, bottom); the range of FRET values at the plateau was wider than for RP_ITC≤7_, mainly reflecting the lower stability of shorter RNA within ITCs. For RP_ITC≤5_, a higher plateau (E^∗^∼0.36) was reached in ∼2 s after NTP addition (Figure 2B, bottom); the range of FRET values at the plateau was as for RP_ITC≤7._. To compare the FRET-based distance changes to structural model predictions, we calculated the corrected FRET efficiencies for the stable scrunched states and their corresponding distances (Figure S1A); while the observed distance decrease upon going from the stable scrunched state of RP_ITC≤4_ to that of RP_ITC≤5_ was similar to the model prediction (ΔR_model_ ∼10 Å; ΔR_exp_ ∼8 Å), the distance decrease for the transition from RP_ITC≤5_ to RP_ITC≤7_ was much smaller than expected (ΔR_model_ ∼9 Å; ΔR_exp_ ∼1 Å), raising the possibility that the main abortive RNAs in RP_ITC≤7_ were shorter than a 7-mer.

To obtain the distribution of short transcripts at our promoter for RP_ITC≤7_, we performed in vitro transcription (Figure 2C; for gel band assignment, see Figure S2A). The results showed that RP_ITC≤7_ synthesized a substantial fraction of 6-nt RNA (5′-AAUUGU-3′), an RNA one nucleotide shorter than expected for this complex. At short incubations (10–20 s, similar to the timescale for the FRET measurements), the 6-nt RNA was the main product and was slowly extended (t_1/2_ ∼20 s) to a 7-mer (5′-AAUUGUG-3′); the 7-mer became the main product in 60 s (Figures S2B–S2D). This behavior is the hallmark of transcriptional pausing. Importantly, the 6-nt RNA was also present for complexes supplied with all NTPs (run-off; Figure 2C, lane 7), showing that the paused complex at 6-nt RNA was an on-pathway intermediate. In contrast, the 7-nt RNA was almost absent in the run-off reaction, showing that extension beyond a 7-mer was efficient, and that there was no significant pausing after synthesis of a 7-mer; we obtained identical results on a *lac*UV5 promoter (which differs from *lac*CONS by not having a consensus −35 and consensus −10/−35 spacer; Figure S3). The prevalence of a 6-mer RNA in ITCs capable of synthesizing a 7-mer was consistent with studies on *lac*UV5 (Brodolin et al., 2004, Carpousis and Gralla, 1980).

### RNAP Pausing during Initial Transcription by Single ITCs

**Figure 3:**
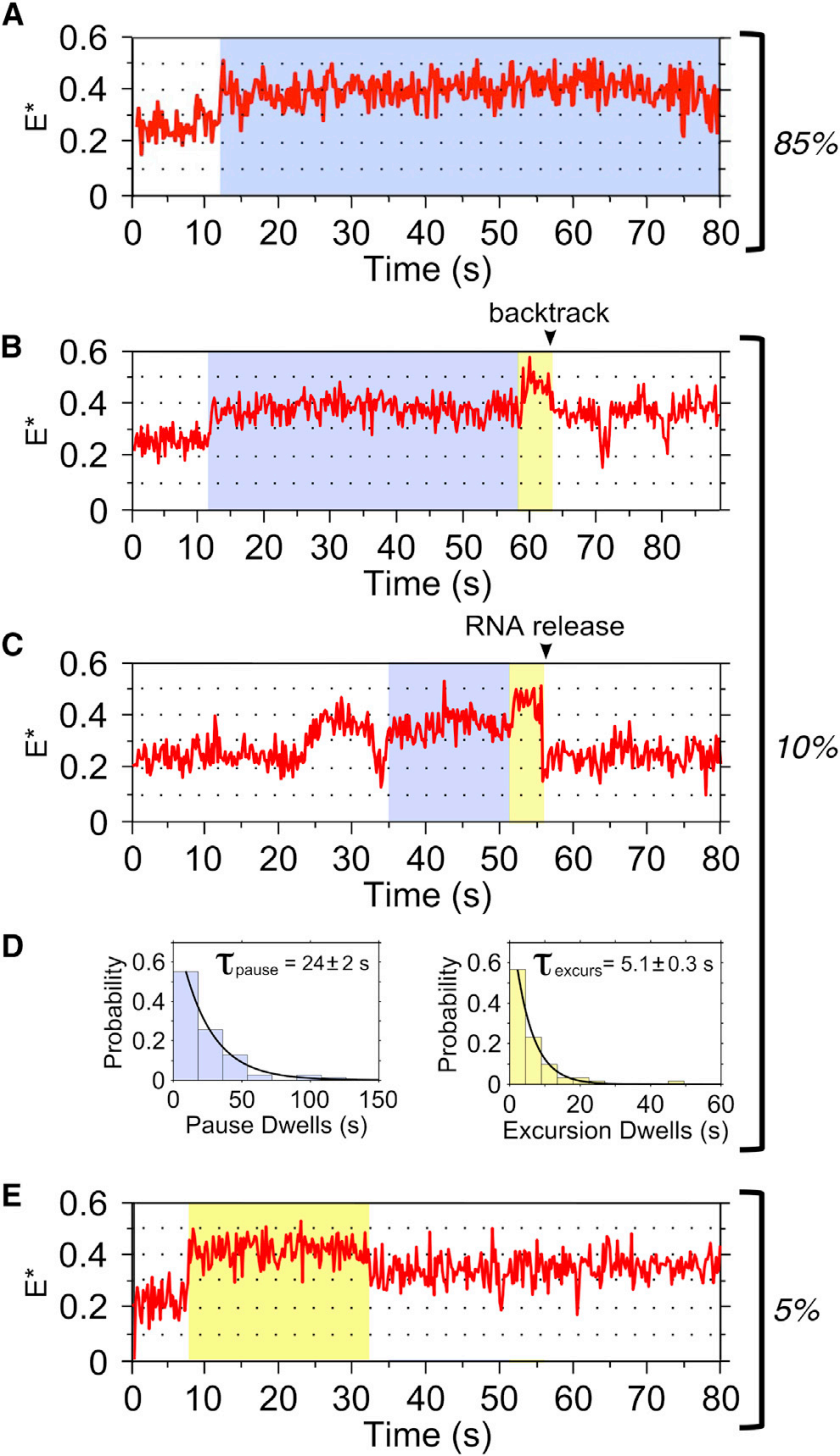
Single-Molecule Transcription by RP_ITC≤7_ Complexes Frame time: 200 ms. (A) Time trace showing an increase to a stable E^∗^∼0.37 state. (B) Time trace showing pausing at E^∗^∼0.37 (highlighted blue), followed by an excursion to the E^∗^∼0.45 state (highlighted yellow). The return to the stable E^∗^∼0.37 is assigned to RNA backtracking. (C) Time trace showing pausing, followed by an excursion to E^∗^∼0.45 (as in B), followed by a return to the RP_o_ baseline (assigned to RNA release). (D) Dwell-time histograms and exponential fits for the paused state (left; n = 84) and the E^∗^∼0.45 state (right; n = 60). (E) Time trace showing no pausing before reaching E^∗^∼0.45, followed by a return (highlighted yellow) to a stable E^∗^∼0.37 state.

To further study the FRET states in RP_ITC≤7_, we examined individual traces. As expected, ∼85% of all traces (221 of 260) showed complexes reaching the long-lived state of E^∗^∼0.37 (Figure 3A). Based on our in vitro transcription results (where the 6-mer accumulates before converting to a 7-mer), we assigned the E^∗^∼0.37 state to a complex with an RNA of 6 nt in length (i.e., RP_ITC6_). We also saw that in ∼15% of the traces (n = 39), an E^∗^∼0.45 state is reached, which we assigned to RP_ITC7,_ a complex containing a 7-mer RNA (the longest RNA synthesized with the nucleotide subset used); in ∼65% of these traces (n = 25), the E^∗^∼0.45 state was reached after a pause at E^∗^∼0.37 for several seconds (Figures 3B and 3C), while in the rest of the traces, the E^∗^∼0.45 state was reached without apparent pausing (Figure 3E). These results, along with our in vitro transcription, suggest that RNAP enters a long-lived paused state after synthesizing a 6-nt RNA, which is then slowly extended to a 7-nt RNA.

To study the pausing kinetics, we plotted the pause-time distribution for molecules that occupied the E^∗^∼0.37 state before the E^∗^∼0.45 state. The distribution fitted well to a single-exponential decay (indicating a single rate-limiting step) with a duration of 24 ± 2 s (Figure 3D, left); this long lifetime suggests that the pause could be rate-limiting for promoter escape.

Once the E^∗^∼0.45 state was reached, the complex either returned to the E^∗^∼0.37 state (Figures 3B and 3E) or the RP_o_ baseline (Figure 3C). On average, the lifetime of the E^∗^∼0.45 state was 5.1 ± 0.3 s (Figure 3D, right). Since RNAP can form 7-mers (Figure 2C), the return to the E^∗^∼0.37 was likely due to RNA backtracking in RP_ITC7_ to the translocational register seen for the 6-mer RNA (see Discussion). Further, the return to the RP_o_ baseline, frequently followed by additional cycling to higher FRET states, is consistent with abortive RNA release.

### Scrunched Complexes Are Stable after Synthesis of a 6-nt RNA

**Figure 4:**
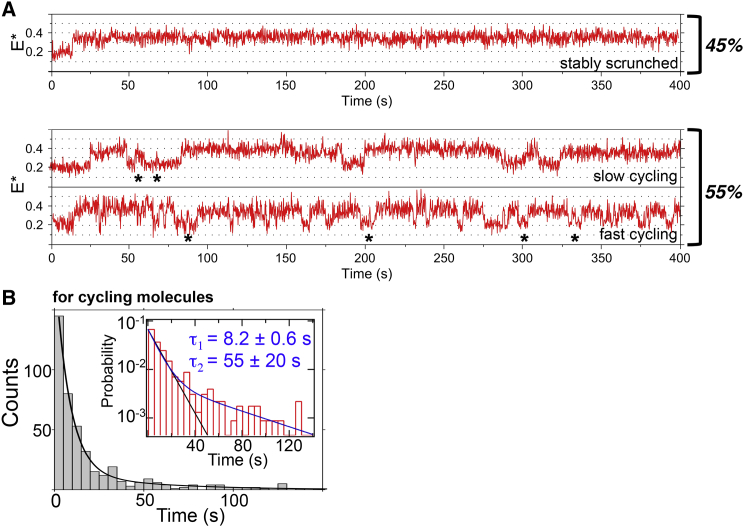
Single-Molecule Transcription by RP_ITC≤7_: Extended Observations Frame time: 200 ms. (A) Time traces of stable scrunched (top) and abortive cycling (middle and bottom) transcribing RP_ITC≤7_. Events that may show short (<5 nt) abortive RNAs being synthesized and released are marked with asterisks. (B) Distribution of scrunched-state dwell times for cycling molecules (n = 445), shown in a linear and semi-log plot (inset). The distribution is fitted well by a short and a long lifetime (∼85% and ∼15% of the events, respectively); a single-exponential fit (black line in inset) fails to account for the population of long-lived dwells. Most short dwells come from fast cycling molecules. See also Figure S3.

We then examined the stability of RP_ITC≤7_ complexes occupying the E^∗^∼0.37 state (RP_ITC6_) by analyzing complexes retaining their FRET pair for >10 min (Figure 4). About 45% of the complexes adopted a single E^∗^∼0.37 state for >120 s (“stably scrunched complexes”; Figure 4A, top). The rest adopted scrunched states for <120 s, followed by a return to the RP_o_ baseline and new rounds of RNA synthesis (“cycling complexes”; Figure 4A, middle and bottom).

To evaluate the stability of scrunched states in cycling complexes, we analyzed the distribution of dwell times in the scrunched state; the distribution exhibited bi-exponential decay kinetics with mean times of t_1_∼8 s and t_2_∼55 s (Figure 4B). The long-lived species is likely to be similar to the stably scrunched complexes. We obtained similar lifetimes for RP_ITC≤7_ complexes formed on a fully double-stranded promoter DNA fragment (Figure S3), showing that the stability of scrunched complexes is unaffected by the mismatch in our pre-melted DNA.

### Region σ3.2 Blocks RNA Extension beyond 6 nt

**Figure 5:**
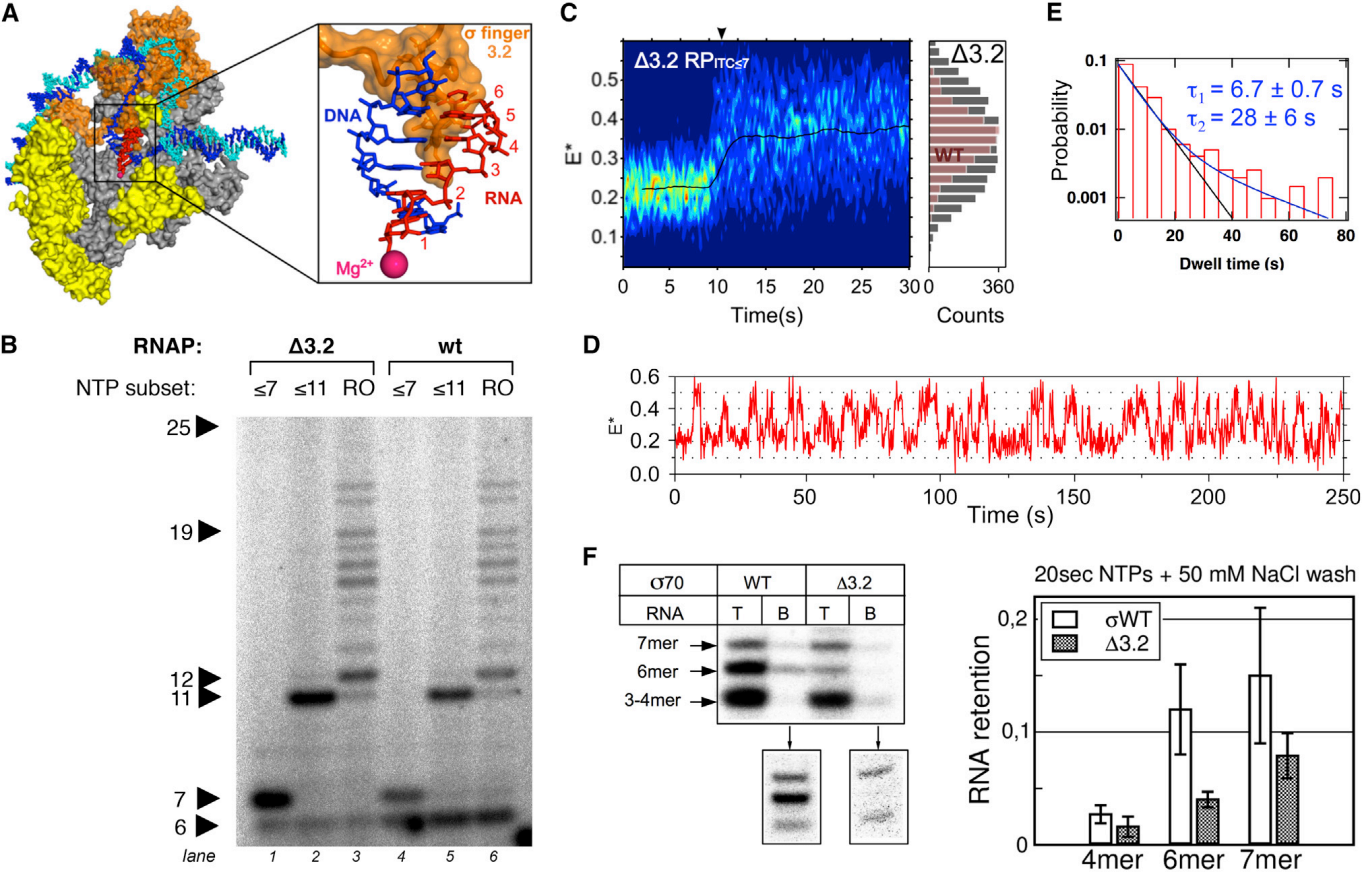
Region σ3.2 Is a Major Determinant of Initiation Pausing (A) Structural model of an ITC highlighting the clash between nascent 6-nt RNA (in red) and σ3.2 (in orange). Colors as in Figure 1A. (B) Comparison of transcription by Δ3.2 versus WT RNAP on *lac*CONS. Lanes 1–3: RNAs produced by Δ3.2 complexes able to synthesize up to 7-nt RNA (lane 1), up to 11-nt RNA (lane 2), and up to a run-off product (a 25-mer; lane 3). Lanes 4–6: RNAs produced by same mixtures as for lanes 1–3, but for WT RNAP. (C) Heatmaps for Δ3.2 complexes in RP_ITC≤7_. Right-side histogram: collapse of all E^∗^ values in the high-FRET plateau (reached at ∼12 s; gray bars). Frame time: 200 ms. The E^∗^ full width at half maximum was ∼0.34 for Δ3.2, ∼2-fold wider than for WT (0.18 ± 0.02, pink bars). (D) Time trace where E^∗^∼0.45 is sampled frequently and without long pauses at E^∗^∼0.37. (E) Dwell-time distributions of Δ3.2 scrunched states (n = 392). (F) Retention of 6-nt and 7-nt RNAs in complexes due to σ3.2 presence. Reactions for RP_ITC<7_ were run for 20 s at 37°C on bead-immobilized RP_o_; reactions were stopped, and complexes were washed and incubated for ∼2 min before gel loading. WT panel: using WT sigma and no washing (“T” lane), in vitro transcription yields 6-mers and 7-mers, as well as unresolved 3/4-mers. As for *lac*CONS, the 6-mer is more abundant than the 7-mer, consistent with pausing at 6-nt RNA. After washing and incubation (“B” lane), little 3/4-mer is retained; in contrast, there is much higher retention of 6-mer and 7-mer RNA. Lower inset: sample from lane B was run in a separate lane and overexposed. Δ3.2 panel: using Δ3.2 and no washing, in vitro transcription yields 6-mers, 7-mers, and unresolved 3/4-mers; as for *lac*CONS, with the 6-mer/7-mer distribution shifted substantially to 7-mer. There is little retention for 3/4-mers and 6-mer in Δ3.2, although there is moderate retention of the 7-mer, likely due to a more stable RNA-DNA hybrid. Right panel: quantitative comparison of RNA retention on bead-immobilized RP_ITC<7_; results reflect mean and SD of four independent experiments.

An explanation for the inability of most RP_ITC≤7_ complexes to rapidly synthesize a 7-nt RNA is the presence of structural elements that block motions for smooth progression from RP_ITC6_ to RP_ITC7_; such elements may also destabilize the RP_ITC7_ state when reached, as suggested by the short dwell in the E^∗^∼0.45 state (Figures 3B, 3C, and 3E). A candidate for this role is σ region 3.2, a part of which forms an unstructured loop (also known as “σ finger”) that partially occupies the RNA exit channel (Basu et al., 2014, Murakami, 2013, Zhang et al., 2012). Based on structural models, the 5′ end of RNA is expected to clash with σ3.2 when the RNA becomes 5- to 6-nt long (Figure 5A; Murakami et al., 2002, Zuo and Steitz, 2015). We thus tested whether σ3.2 affects RNA extension beyond a 6-mer; we also hypothesized that deleting σ3.2 would increase the yield of 7-nt RNAs produced by RP_ITC≤7_ and eliminate pauses en route to E^∗^∼0.45.

To test our hypotheses, we studied complexes formed using a mutant RNAP lacking part of σ3.2 (Δ3.2, lacking residues 513–519; Kulbachinskiy and Mustaev, 2006). The Δ3.2 mutant is expected to have a more accessible RNA exit channel and weaker interactions with the template strand. Indeed, Δ3.2 RP_ITC≤7_ complexes synthesized mainly a 7-nt RNA (Figure 5B, lane 1), as opposed to wild-type (WT) complexes, which synthesized similar amounts of a 6-nt and 7-nt RNA (Figure 5B, lane 4). Further, upon NTP addition that allows Δ3.2 RNAP to form an RNA of up to 11 nt in length (Figure 5B, lane 2), or a run-off product (a 25-nt RNA; Figure 5B, lane 3), the 6-nt RNA was greatly reduced (but not eliminated) relative to the amount for WT complexes, which synthesize a 6-mer as their main short transcript (Figure 5B, lanes 5 and 6). These results establish σ3.2 as a major pausing determinant after RNAP synthesizes a 6-mer on *lac*CONS. Notably, the fact that the 6-nt RNAs are not eliminated for Δ3.2 under all conditions (RP_ITC≤7_, RD_e11_, and run-off) points to the presence of additional pausing determinants.

We performed similar comparisons using smFRET on RP_ITC≤7_ complexes and found major differences between the Δ3.2 and WT RNAP complexes. Heatmaps (Figure 5C) showed that Δ3.2 complexes sample higher FRET states more readily than WT (∼17% ± 5% of Δ3.2 states show E^∗^>0.45 versus ∼6% ± 2% for WT; mean ± SD); this is despite the fact that Δ3.2 complexes with E^∗^>0.3 are less stable and dissociate quickly, broadening the FRET distribution after NTP addition (E^∗^ full width at half maximum was ∼0.34 for Δ3.2 and ∼0.18 for WT; see also Figure 5C).

We then compared time traces of Δ3.2 and WT RNAP complexes (Figure 5D). First, Δ3.2 RP_ITC≤7_ complexes reached the E^∗^∼0.45 state more often than WT RP_ITC≤7_ complexes (72 of 219 molecules for Δ3.2, i.e., 33% ± 5% of all transitions versus 15% ± 5% for WT; mean ± SD). Second, the vast majority of Δ3.2 complexes that did reach the E^∗^∼0.45 state (90% of 47 molecules) did so without an apparent pause at E^∗^∼0.37 (Figure 5D); the same number for WT was only ∼30%. Third, there was a large decrease in the fraction of stably scrunched molecules (15% ± 7% for Δ3.2 complexes versus 46% ± 5% for WT complexes). The scrunched states in the Δ3.2 RP_ITC≤7_ complex were also significantly less stable, as judged by the ∼20% and ∼50% decrease in the fast and slow scrunched-state lifetimes, respectively (Figure 5E). This observation suggests that σ3.2 acts not only as a barrier to the 6-mer extension, but also contributes to the stable attachment of the 6-mer within RP_ITC≤7_.

To further study the attachment of 6-mer to RP_ITC≤7_ complexes and its dependence on σ3.2, we performed in vitro transcription on bead-immobilized complexes and examined the profile of RNAs retained by the complexes after a 2 min wash (Figure 5F). Approximately 14% of the total 6-nt and 7-nt RNA is retained in the complex after the wash, which implies an average RNA retention lifetime of ∼1 min. Identical experiments for Δ3.2 showed 3-fold lower retention for the 6-mer (and 2-fold for the 7-mer), likely due to loss of σ3.2 interactions with parts of the transcription complex that control scrunching and RNA release. These results establish that a substantial portion of the accumulated 6-mer seen on transcription gels is due to RNA stably attached to the transcription complex, as opposed to being released quickly as abortive products.

### Promoter Escape Involves Additional RNAP Pausing

**Figure 6:**
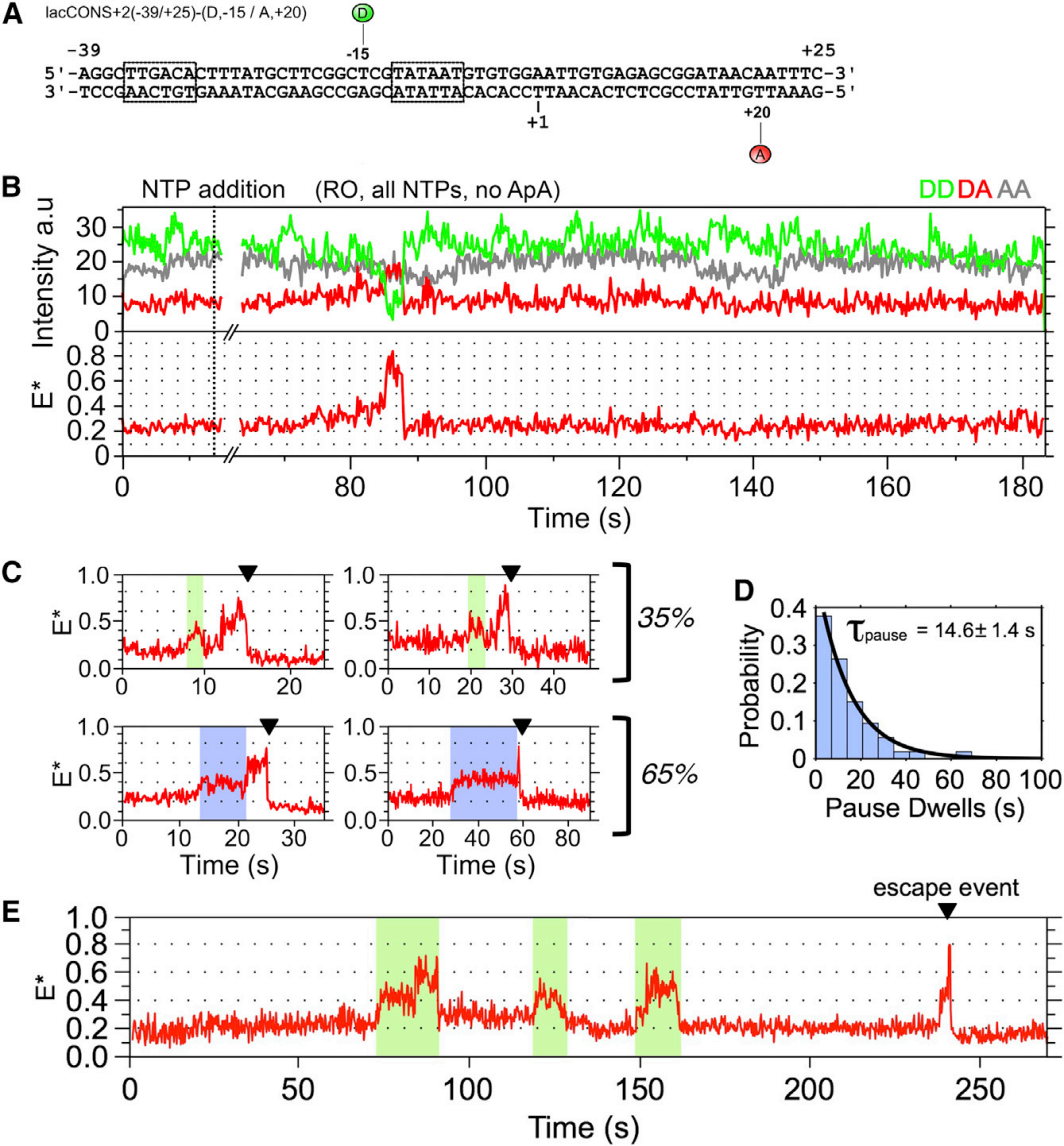
Initiation Pausing on the Path to Promoter Escape (A) Promoter DNA fragment used. (B) Time trace showing FRET changes consistent with escape. We added 200 μM ATP and 100 μM UTP, GTP, and CTP. The escape event was marked by the sharp FRET decrease from the maximum FRET state to the baseline (∼88 s). No significant E^∗^ change is observed after escape during the remaining ∼100 s. (C) Common behaviors consistent with escape. Top panels: example of escapes (marked by black arrowhead) preceded by a clear abortive cycle (green highlight). Bottom panels: examples of escapes preceded by a pause at E^∗^∼0.37 (blue highlight). (D) Dwell-time distribution of pauses in (C) (n = 130). (E) Time trace showing three abortive cycles (green highlight) followed by a cycle consistent with escape. See also Figure S4.

All FRET experiments so far were on complexes synthesizing RNAs of up to 7 nt in length. To place our studies in the context of the entire initial transcription up to promoter escape, we performed smFRET on surface-immobilized complexes provided with all four NTPs. Based on our molecular modeling and the DNA conformational changes during escape (Kapanidis et al., 2005b, Mukhopadhyay et al., 2001), we expected a further FRET increase beyond the E^∗^∼0.45 state prior to escape due to additional DNA scrunching. This increase was expected to reach a maximum at the point of escape, leading to a FRET decrease when RNAP breaks its promoter contacts and translocates forward by a turn of DNA. After this transition, we expected FRET to stay low (at levels similar to that for RP_o_). To avoid any potential interference with the re-annealing of the upstream region of the transcription bubble during escape, we used homoduplex DNA (Figure 6A).

Several time traces showed the pattern expected for escape (Figures 6B and 6C) and exhibited four main features: first, upon NTP addition, complexes displayed a FRET increase to a maximum E^∗^of ∼0.6–0.8; no such states were seen in RP_ITC≤7_. Second, in ∼50% of such traces, the FRET change included a pause at E^∗^ of 0.35–0.4 before reaching E^∗^ > 0.6 states (Figure 6C, bottom); the pause lasted for 15 ± 1 s (Figure 6D), similar to that observed in RP_ITC≤7_, and is clearly not a promoter-proximal paused state (Nickels et al., 2004), since such a state would appear only after formation of a low-FRET state (matching the RP_o_ baseline), something not observed in our traces. The remainder 50% showed no clear pause, but part of this population almost certainly includes pauses too short to capture given our temporal resolution (200 ms). Third, once the E^∗^∼0.6–0.8 FRET state was reached, the complexes remained at that state for ∼8 s (Figure S4A) prior to returning to the baseline. Fourth, after returning to the baseline, no subsequent FRET events were observed within our observation span; however, since any observation of cycling is limited by bleaching, we cannot unequivocally define the point of escape.

The long dwell at E^∗^∼0.35–0.4 confirmed that the paused state in RP_ITC≤7_ is a true intermediate on the path to escape. Finally, the long dwell (∼8 s) in the maximum FRET state corresponds to a state occupied just before the point where RNAP breaks its promoter interactions during escape; we refer to this pause as the “escape pause.”

Most molecules reaching the maximum FRET state (65%) do not go through cycling involving synthesis of >4-nt RNAs (Figure 6C, bottom; we cannot detect all abortive RNAs shorter than 5 nt since they do not stably attach to ITCs). The remainder 35% reached the maximum FRET state after cycling (Figure 6C, top panels; Figure 6E). Notably, most RNAP molecules (∼70%) did not escape, despite being provided the full set of NTPs at sufficiently high concentrations (≥100 μM); instead, they appear to be locked in abortive transcription, with ∼90% resembling RP_ITC≤7_ (Figure S4B).

## Discussion

### A Long Transcriptional Pause on a Promoter Rate-Limited in Initial Transcription

Our results establish that initial transcription on *lac* promoter is not a continuous process, but is interrupted by a long pause (“initiation pause”) after RNAP synthesizes a 6-nt RNA. The observation of high levels of a 6-nt RNA (along with the absence of a 5- or 7-nt RNA) in the reaction with all NTPs agrees with early observations on *lac*UV5 (Carpousis and Gralla, 1980, Munson and Reznikoff, 1981). The paused initiation complex on *lac* promoters is thus a true intermediate on the path to elongation.

Due to its long lifetime (∼20 s), the initiation pause can be rate-limiting for initial transcription. The pause is substantially longer than open-complex formation at *lac*CONS (∼3 s; Revyakin et al., 2004), and comparable to open-complex formation at *lac*UV5 (∼10 s at 37°C and ∼30 s at 25°C; Buc and McClure, 1985). The initiation pause is comparable to pauses in elongation, such as promoter-proximal pauses (∼30 s at 200 μM NTPs on *lac*; Nickels et al., 2004), and the “elemental” pause (1.5–10 s, depending on GTP concentration; Larson et al., 2014; ∼10 s; Hein et al., 2014).

### Promoter Dependence of Initiation Pausing

Apart from *lac*, many other promoters are likely to display initiation pausing. For example, Tn5 promoter, also rate-limited in initial transcription, showed accumulation of a 6-nt RNA (Munson and Reznikoff, 1981). Further, removal of σ3.2 caused a marked change in the pattern of short RNAs both on a T7A1cons and a *gal*P1 promoter (Pupov et al., 2014); the longest RNAs eliminated by σ3.2 removal on T7A1cons likely reflect paused ITCs with RNAs equivalent to 5- and 6-nt RNA. The excellent agreement with the length of 6-nt RNA seen on our *lac* promoter supports the presence of initiation pauses in these promoters.

There are, however, promoters linked to limited short RNA transcription prior to escape (e.g., T5N25, *rrn*B); such promoters should exhibit less pausing, whereas promoters limited in initial transcription should exhibit significant initiation pausing. This promoter dependence also implies that although σ3.2 is a major pausing determinant, there are additional, DNA-sequence-dependent determinants that modulate the transition from 6- to 7-nt RNA; this is supported by the fact that σ3.2 removal did not eliminate 6-nt RNA accumulation on *lac*CONS (Figure 5B). It is likely that some of these sequence determinants are present in the initial transcription sequence, since it can drastically change the profile of abortive transcripts (Hsu et al., 2006). Consistent with this, we showed that altering the DNA sequence at positions +6 and +7 to remove a short sequence element (Y^−1^G^+1^, also a major determinant of elongation pausing; Vvedenskaya et al., 2014, Larson et al., 2014) significantly reduces initiation pausing at *lac* and on many promoters carrying the sequence element (Bauer et al., 2016).

### Possible Roles of Initiation Pausing

Initiation pausing can modulate the rate of promoter escape and RNA synthesis. Initiation pausing can also act as a timing delay that increases the spacing between RNAP molecules in elongation, affecting pausing in elongation (Epshtein and Nudler, 2003) and transcription-translation coordination. For some promoters, the combination of multiple rate-limiting steps of similar timescale (e.g., for *lac* promoter, where promoter melting, initiation pause, and promoter-proximal pause last 20–30 s each; Buc and McClure, 1985, Nickels et al., 2004) can turn an exponential distribution of transcription times (i.e., as for a single rate-limiting step) to a distribution with a longer and less variable time delay between RNAPs leaving the promoter. Initiation pausing may also provide more opportunities for regulatory proteins and small molecules to bind ITCs and modulate transcription.

### Region σ3.2 Controls Pausing by Transiently Blocking RNA Extension beyond 6 nt

Our work establishes region σ3.2 as a major determinant for initiation pausing and as the structural element that controls the position of initiation pausing. Region σ3.2 interacts with the template strand (positions −3 and −4) and blocks the RNA exit path by clashing with the 5′ end of nascent RNA (Basu et al., 2014, Kulbachinskiy and Mustaev, 2006, Murakami, 2013, Zhang et al., 2012); σ3.2 has also been shown to be a major determinant of abortive initiation (Murakami et al., 2002). Partial removal of σ3.2 changes the distribution of short RNAs (e.g., decreasing the levels of 5- to 9-nt RNAs) at the T7A1cons promoter (Kulbachinskiy and Mustaev, 2006, Pupov et al., 2014). Such changes led to proposals that σ3.2 hinders RNA extension, while its removal allows extension of RNAs that would otherwise abort (e.g., 5- to 9-nt RNAs on T7A1cons).

Our results show σ3.2 indeed acts as the protein element that sets the stage for pausing at RP_ITC6_; we suggest that the presence of σ3.2 along the path of growing RNA provides an initial time window (linked to σ3.2 repositioning) that allows RNAP to enter paused states, the stability of which is governed by a complex landscape of determinants, including DNA sequence. In short, σ3.2 is the RNAP structural element that *enables* initiation pausing (and consequently, regulation) at the 6-to-7 transition.

Our results also suggest that σ3.2 stabilizes the scrunched conformation in RP_ITC6_, with stabilization seen first when RNA reaches 5 nt in length. One possibility for the stabilization is that the 5′ end of RNA interacts with σ3.2, as suggested by ITC structures (Basu et al., 2014, Zuo and Steitz, 2015); since the structures showed σ3.2 in slightly different conformations, these conformations may be linked with different pause-recovery kinetics. Interactions between template and σ3.2 may also prevent lateral movements of the template strand that would otherwise allow RNA to backtrack and be released more easily (see Discussion on backtracking; Pupov et al., 2014); consistent with this, a Δ3.2 mutant exhibits faster bubble dynamics in RP_o_ (D.D. and A.N.K., unpublished data).

### Backtracking and Abortive Release Mechanism

Our FRET results on RP_ITC7_ revealed transitions consistent with scrunching relaxation by RNA backtracking, since the relaxed state matches the FRET signature of the paused state in RP_ITC6_, which is likely to be in its pre-translocated state. In the backtracked RP_ITC7_ state, RNAP is inactive, since its active site is blocked by the 3′ end of RNA; this state also leads to RNA loss. These series of transitions suggest that the backtracked state is an intermediate on the path to RNA release. RNA backtracking in initiation is supported by reports showing that transcript-cleavage factor GreA (which cleaves the 3′ end of RNA in backtracked elongation complexes to generate new extensible 3′ ends) alters the abortive products on T7A1 and T5N25anti (Feng et al., 1994, Hsu et al., 1995), as well as by in vivo work suggesting that the main GreA role is to relieve transcriptional arrest at specific promoters before promoter clearance (Stepanova et al., 2007). These findings support a model wherein short RNAs are displaced from the active center in a backward direction, form backtracked states (wherein the 3′ end of RNA frays from the template and enters the secondary channel), and get released (Feng et al., 1994, Hsu, 2009, Hsu et al., 1995, Stepanova et al., 2007).

### Initial Transcription and Promoter Escape

We also observed DNA conformational changes occurring between the 6-mer pause and promoter escape. Notably, we observed a pause just before escape (“escape pause”), where the maximum scrunching is expected to be reached; this pause may reflect destabilization of contacts between σ region 4 (σ4) and the −35 element (Vassylyev et al., 2002) or the last stage of σ3.2 displacement from the RNA exit channel, an event that affects σ4-promoter interactions (Mekler et al., 2002, Murakami and Darst, 2003, Murakami et al., 2002, Vassylyev et al., 2002). The escape pause presents another rate-limiting step with regulation potential.

### Heterogeneity of ITCs

Our results showed that active complexes exhibit heterogeneity, since ITCs imaged under identical conditions displayed varying tendencies for abortive cycling (Figure 4A). The heterogeneity was long lived, with “stably scrunched” or “cycling” behaviors persisting for >10 min. Such functional heterogeneity has been seen in elongation (Herbert et al., 2006) and may reflect the presence of moribund abortive complexes (Hsu, 2002, Kubori and Shimamoto, 1996) that could underpin a mode of regulation; e.g., regulatory molecules or different promoters may affect the distribution between behaviors, altering the probability of producing full-length RNA. The heterogeneity source is unclear, but it may reflect static conformational heterogeneity between molecules, as well as compositional differences between molecules, due to translation errors or chemical changes occurring either in vivo or during RNAP preparation, as suggested for elongation (Larson et al., 2011).

### A Working Model for Initial Transcription

**Figure 7:**
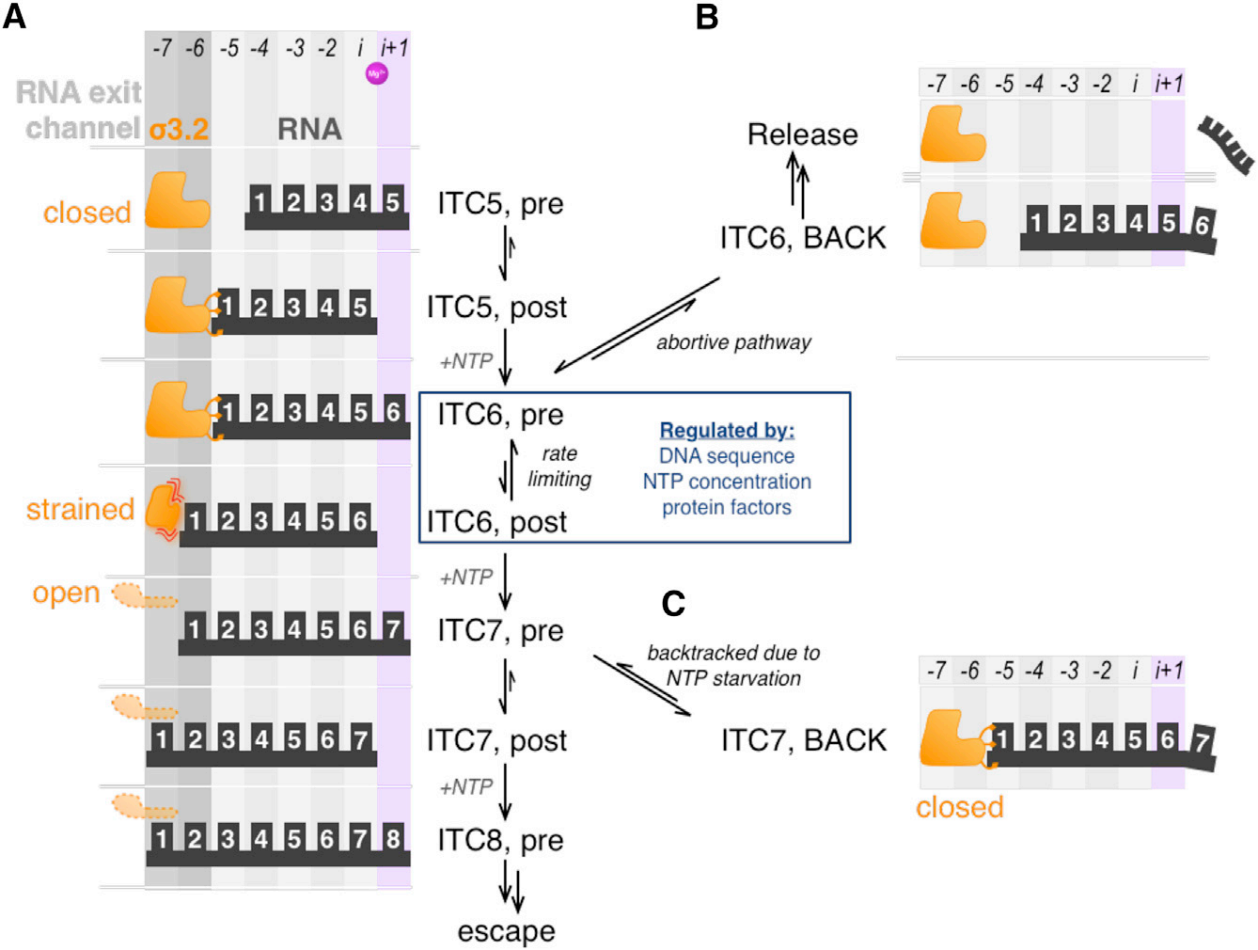
A Working Model for Initial Transcription (A) Productive path for initial transcription. Colored columns show translocational registers adopted by growing RNA (in black). Binding site for incoming NTP is in light purple; σ3.2 loop is shown in three putative conformations (in orange). The translocational equilibrium for RP_ITC6_ is controlled by several regulatory factors that modulate the lifetime of paused states arising from a pre-translocated RP_ITC6_. (B) Abortive path for initial transcription, branching from the pre-translocated RP_ITC6_ state of the productive path. (C) Path for the formation of stable backtracked scrunched states, branching from the pre-translocated RP_ITC6_ state of initial transcription during NTP starvation that limits RNA synthesis to 7 nt in length.

Based on our findings and existing literature, we present a working model for initial transcription that includes initiation pausing as a regulatory checkpoint controlled by structural, sequence, and environmental factors (Figure 7). While the model focuses on *lac*, many features should apply to most bacterial promoters.

Initial transcription starts with synthesis of RNAs 2–4 nt in length, accompanied by increasing scrunching; these products dissociate quickly (Carpousis and Gralla, 1980). When the RNA reaches 5 nt in length, it is stabilized in RP_ITC5_, most likely at its post-translocated state. This frees the i+1 site at the active center for binding the next complementary NTP, which is incorporated quickly to form a pre-translocated RP_ITC6_ (as seen in our results and in a complex resembling RP_ITC6_; Basu et al., 2014). The presence of σ3.2-template interactions limits initial scrunching to 4 nt (i.e., up to the initiation pause) in the template and non-template strands. The 5′ end of the 6-nt RNA clashes with σ3.2, hindering template/RNA translocation from the pre- to post-translocated state.

At this point, and in a way akin to “ubiquitous” pausing in elongation (Herbert et al., 2006), the complex enters an off-pathway paused state. The lifetime of pausing is modulated by several determinants (such as DNA sequence, nucleotide identity and concentration, and protein factors); this multi-partite modulation effectively controls the kinetics of the transition from the pre- to post-translocational register of RP_ITC6_ and regulates initial transcription.

At *lac*, the overall context biases the translocational balance toward a pre-translocated RP_ITC6_ and a paused state lasting for 15–25 s. In productive initial transcription, GTP binds to a transiently sampled post-translocated state of RP_ITC6_ and extends RNA to a 7-mer. Although our results point to the translocation step being rate-limiting, we cannot exclude that NTP binding and incorporation may also be affected, as in pauses without backtracking (Kireeva and Kashlev, 2009). The formation of a 7-mer stabilizes RP_ITC7_ and allows translocation to the post-translocated state, where the RNA exit channel entrance is kept open by the 5′ end of RNA. Ultimately, σ3.2 is displaced by the growing RNA, weakening σ^70^-promoter contacts and driving promoter escape. The growing RNA also severs the contacts of σ3.2 with the template, allowing the template to scrunch further up to promoter escape. The evidence for backtracking in the case of NTP starvation (due to the use of NTP subsets) also identifies the secondary channel as the likely RNA release route.

### Relevance to Other Transcription Systems

Since the negative charge of σ3.2 is highly conserved in alternative σ factors, initiation pausing may be present in non-σ^70^ bacterial promoters (Pupov et al., 2014). The conservation of the σ3.2 loop structural feature in eukaryotes and archaea raises the possibility of initiation pausing in a diverse range of organisms, e.g., due to the TFIIB B-finger (Sainsbury et al., 2013) or a similar structure in archaeal TFB; the latter has already been shown to increase abortive transcription when added to a transcribing archaeal RNAP (Werner and Weinzierl, 2005).

## Experimental Procedures

### DNA, RNAP, and RP_o_ Preparation

Labeled oligos were purchased from IBA. WT RNAP core from *E. coli* with a His-tag at the β′ C terminus was prepared as described (Belogurov et al., 2007). WT and mutant σ^70^ lacking residues 513–519 (Δ3.2) were purified as described (Kulbachinskiy and Mustaev, 2006). WT and Δ3.2 holoenzymes were prepared by incubating 50 nM RNAP core with 250 nM σ^70^ for 30 min at 33°C. RP_o_ was formed by incubating RNAP holoenzyme with DNA followed by heparin challenge (Kapanidis et al., 2006). For rifampicin experiments, 250 nM rifampicin was incubated with RNAP for 30 min at 33°C before DNA was added.

### In Vitro Transcription

Reactions were performed as described (Cordes et al., 2010, Robb et al., 2013) with modifications to mimic our smFRET experiments. Reactions were initiated by mixing 1 μL RP_o_ with a 4 μL mix containing 4 U RNAsin, 0.1 mg/mL heparin, and the relevant NTP mixture in 1× KG7 buffer (40 mM HEPES-NaOH [pH 7], 100 mM potassium glutamate, 10 mM MgCl_2_, 100 μg/mL BSA, 1 mM DTT, and 5% glycerol). NTPs and ApA were added at 80 and 500 μM, respectively. Reactions were supplemented with [α^32^P]UTP (0.6 μCi/μL, PerkinElmer), incubated for 10–60 s at 21°C, stopped by 7.5 μL of 1 M HCl, and neutralized with Tris/EDTA (Malinen et al., 2015). The reactions were precipitated and kept at −20°C. Pellets were dried, dissolved in loading dye, and incubated for 4 min at 95°C before gel electrophoresis and autoradiography.

For transcription on beads, RNAP was assembled in 10 μL transcription buffer (TB) (40 mM HEPES [pH 8.0], 50 mM NaCl, 5 mM MgCl_2_, and 5% glycerol) and incubated with 10 μL Ni^2+^ agarose beads; samples were centrifuged and 6 μL TB was discarded. One microliter *lac*UV5 DNA was added and incubated for 10 min at 37°C. Transcription was initiated by 1 μL of 5 mM ApA; 2 μL of 250 μM GTP, UTP (to 31 μM final), and 0.6 μCi [^32^P]-UTP per reaction; and incubation for 20 s at 37°C. Reactions were stopped by washing the complexes; the supernatant was discarded and Ni beads were supplemented with stop solution. Samples were incubated for 2 min at 65°C before being loaded on a PAGE denaturing gel.

### Single-Molecule FRET

TIRF experiments with alternating-laser excitation (Kapanidis et al., 2004) were performed on a custom microscope (Holden et al., 2010). To immobilize RP_o_, 10 nM biotinylated penta-His antibody was incubated for 10 min on a neutravidin-coated surface; unbound antibodies were removed, and 1 nM RP_o_ was added and incubated for 5 min. Once RP_o_ was immobilized, KG7 imaging buffer (40 mM HEPES-NaOH [pH 7], 100 mM potassium glutamate, 10 mM MgCl_2_, 1 mm DTT, 100 μg/mL BSA, 5% glycerol, and 2 mM Trolox) and an oxygen scavenging system (1 mg/mL glucose oxidase, 40 μg/mL catalase, and 1.4% w/v D-glucose) were added.

To form RP_ITC_ synthesizing RNAs up to N nt in length (RP_ITC≤N_), NTP reaction mixtures were added manually during acquisition; unless stated otherwise, the final NTP concentration was 80 μM. For RP_ITC≤4_, the NTP mixture consisted of imaging buffer plus UTP. For RP_ITC≤5_, 3′ dGTP (TriLink BioTechnologies) was added to RP_ITC≤4_ mixture. For RP_ITC≤7_, GTP was added to RP_ITC≤4_ mixture. For promoter escape, the imaging buffer was supplemented with ATP at 200 μM, and UTP, GTP, and CTP at 100 μM.

Fluorescence intensities were extracted using *twoTone* (Holden et al., 2010), and the uncorrected FRET efficiency (E^∗^) was calculated as described (Pinkney et al., 2012). To select traces, we used well-defined criteria (see Supplemental Information). The dwell times of scrunched states were extracted via hidden Markov modeling (HMM) analysis (Le Reste et al., 2012) and fitted with exponentials to extract dwell times.

For extended protocols, see Supplemental Experimental Procedures.

## Author Contributions

A.N.K. conceived and supervised the project. A.N.K., D.D., D.L.V.B., and K.B. designed experiments. D.D., L.F., N.R., and P.Z. performed microscopy measurements. L.C.H. and K.G. performed preliminary microscopy experiments. D.D., D.L.V.B., G.E., K.G., N.R., P.Z., and A.N.K. performed data analysis. D.L.V.B., N.R., A.T., and Z.M. performed biochemical assays. D.D., D.L.V.B., and A.N.K. wrote the manuscript.

## Supplemental Information

Document S1. Supplemental Experimental Procedures and Figures S1–S4

Document S2. Article plus Supplemental Information
