# Supplementary material for: RNA Polymerase Pausing during Initial Transcription
Source: Mol Cell. 2016 Sep 15;63(6):939–50. doi: 10.1016/j.molcel.2016.08.011 (PMC5031556; doi:10.1016/j.molcel.2016.08.011)
Supplement: Document S1. Supplemental Experimental Procedures and Figures S1–S4 [file mmc1.pdf]

Molecular Cell, Volume *63*

Supplemental Information

RNA Polymerase Pausing

during Initial Transcription

Diego Duchi, David L.V. Bauer, Laurent Fernandez, Geraint Evans, Nicole Robb, Ling Chin Hwang, Kristofer Gryte, Alexandra Tomescu, Pawel Zawadzki, Zakia Morichaud, Konstantin Brodolin, and Achillefs N. Kapanidis

SUPPLEMENTAL EXPERIMENTAL PROCEDURES

DNA and protein preparation. Oligonucleotides labelled with Cy3B and ATTO647N were purchased from IBA Life Sciences (Germany). WT RNAP core from *E. coli* with a His-tag at the C-terminal end of the β' subunit was prepared as described (Belogurov et al., 2007). WT σ^{70} and mutant σ^{70} lacking residues 513-519 (Δ3.2) were both purified as described (Kulbachinskiy and Mustaev, 2006; Tupin et al., 2010). The N-terminal 6xHis tag was removed by thrombin cleavage kit. WT and Δ3.2 holoenzymes were prepared by incubating 50 nM RNAP core with 250 nM σ^{70} for 30 min at 33°C.

RP_O formation. RP_O was formed as previously described (Kapanidis et al., 2006; Margeat et al., 2006). Briefly, 10 nM of the promoter DNA fragment was incubated with 50 nM *E. coli* RNAP holoenzyme in KG7 buffer consisting of 40 mM HEPES-NaOH (pH 7), 100 mM potassium glutamate, 10 mM $MgCl_2$, 100 µg/mL BSA, 1 mM DTT, and 5% glycerol to give a final volume of 20 µL. The mixture was incubated at 37°C for 15 min, after which 1 mg/mL heparin sepharose (GE Healthcare) was added to disrupt non-specific RNAP-promoter DNA complexes. The mixture was incubated at 37°C for 30 s and then centrifuged to remove sepharose beads; subsequently, 13 µL of the supernatant was removed and transferred to a pre-warmed tube and incubated for a further 20 min at 37°C. For rifampicin experiments, 250 nM rifampicin was incubated with RNAP for 30 min at 33°C before DNA was added. Then, we followed the RP_O formation protocol described above.

***In vitro* transcription (IVT) reactions**. *In vitro* transcription reactions were performed as described (Cordes et al., 2010; Robb et al., 2013), with minor modifications designed to mimic our smFRET experimental conditions. RP_O was formed with 50 nM promoter DNA and 250 nM holoenzyme in 1x KG7 buffer. *In vitro* transcription reactions were initiated by mixing 1 µL of the RP_O mixture with 4 µL reaction mix containing 4 units of RNAsin (Promega, USA), 0.1 mg/mL heparin, and the relevant NTP mixture in 1x KG7 buffer. Details of the NTP mixtures are given in the relevant figure captions. In all cases however, NTPs were added to a final concentration of 80 µM, and ApA was added to a final concentration of 500 µM. Reaction mixes were supplemented with [α^{32}P]UTP (10 µCi/µL, PerkinElmer) and was added at 0.6 µCi/µL. Reactions were incubated for 10-60 s at 21°C, and stopped by the addition of 7.5 µL of 1M HCl, which was then neutralized with Tris/EDTA (Malinen et al., 2015). The reactions were precipitated using glycogen as a carrier (Hsu, 2009) and kept overnight at -20 ºC. Pellets were dried and then taken up in 10 µL of loading dye (90% formamide, 10 mM EDTA, amaranth dye). Mixtures were incubated for 4 min at 95°C before being loaded on a 7 M urea, 20% polyacrylamide sequencing gel and visualized by autoradiography.

***In vitro* transcription on bead-immobilized transcription complexes.** The RNAP holoenzyme was assembled by mixing 200 nM core RNAP and 1 µM σ subunit in 10 µl TB (40 mM HEPES pH 8.0, 50 mM NaCl, 5 mM $MgCl_2$; 5% glycerol) and incubating for 5 min at 37°C. The mixtures were incubated with 10µL Ni^{2+} agarose beads for 5 min at 25°C with shaking at 800 rpm. Samples were centrifuged and 6 µL of TB was discarded. One µl of a 116-bp *lac*UV5 promoter fragment (promoter positions -59 to +58, see Morichaud et al., 2016) was added to 40 nM final and incubated for 10 min at 37°C. Transcription was initiated by adding 1 µl 5 mM ApA, followed by 2 µl of 250 µM GTP, UTP (to 31 µM final) and 0,6 µCi [^{32}P]-UTP per reaction, and incubation for 20 s at 37°C. Reactions were stopped by washing the complexes with 0.5 ml of TB for 30 s; after centrifugation (30 s), the supernatant was discarded and Ni-agarose beads were supplemented with stop solution; the time from the end of the transcription reaction to the addition of stop solution was ~2 min. The sample volumes in the "input" and "wash" reactions were adjusted to be equal. Samples were then incubated for 2 min at 65°C before being loaded on a 24% PAGE-7M urea denaturing gel. Gels were scanned with Typhoon 9400 Imager (GE Healthcare) and quantified using ImageQuant software.

Instrumentation. Single-molecule TIRF experiments were performed on a custom built objective-type TIRF microscope. A green (532 nm; Cobolt Samba) and a red (635 nm; Cube Coherent) laser were combined using a dichroic mirror and then coupled into a fiber optic cable. The fiber output was focussed into the back focal plane of the objective (100× oil-immersion, numerical aperture 1.4, Olympus) and displaced off the optical axis such that the laser light was incident at the slide-solution interface at an angle greater than the critical angle, creating an evanescent wave. ALEX (Kapanidis et al., 2004) was implemented by directly modulating the two lasers. Data was acquired at different alternation rates depending on the experiment. Alternation rates are given in figure legends. Fluorescence emission was collected from the objective and separated from the excitation light by a dichroic mirror (545 nm/650 nm, Semrock) and clean-up filters (545 nm LP, Chroma; and 633/25 nm notch filter, Semrock). The emission signal was focussed on a slit to crop the image and then spectrally separated (using a 630 nm DRLP dichroic; Omega) into donor and acceptor emission channels, which were focused side-by-side onto an EMCCD camera (Andor iXon 897). A CRIFF (ASI) autofocus system was used to ensure focus stability during experiments**.**

Single-molecule FRET experiments. A 10 nM solution of biotinylated penta-His antibody (Qiagen) was incubated for 10 min on a surface coated with NeutrAvidin (Thermo Scientific) to allow binding. After the incubation, excess unbound antibodies were washed away, and a 1 nM solution of pre-formed RP_O was added and incubated for 5 min. Binding was monitored until ~60 molecules were deposited on the surface, and excess unbound complexes were washed away. Once RP_O was immobilized onto the antibody-coated surface, KG7 imaging buffer consisting of 40 mM HEPES-NaOH (pH 7), 100 mM potassium glutamate, 10 mM $MgCl_2$, 1 mm DTT, 100 µg/mL BSA, 5% glycerol, 2 mM UV-treated Trolox, and an oxygen scavenging system (1 mg/mL glucose oxidase, 40 µg/mL catalase, 1.4% w/v D-

glucose) was added. For abortive initiation, the imaging buffer was supplemented with 500 μM ApA dinucleotide (Kapanidis et al., 2006; Margeat et al., 2006). Where indicated, the imaging buffer was supplemented with 500 nM rifampicin.

To form RP_{ITC} engaged in abortive synthesis of RNA products up to N-nt in length ($RP_{ITC\leq N}$), an NTP reaction mixture was added to the observation chamber manually during data acquisition. The NTP reaction mixture varied for different experiments; however, unless stated otherwise, the final concentration of NTPs in the observation chamber was 80 μM. For $RP_{ITC\leq 4}$ experiments the NTP reaction mixture consisted of the imaging buffer supplemented with UTP. For $RP_{ITC\leq 5}$ experiments, 3'-dGTP (TriLink BioTechnologies) was added to the $RP_{ITC\leq 4}$ mixture. For $RP_{ITC\leq 7}$ experiments, GTP was added to the $RP_{ITC\leq 4}$ mixture.

For promoter-escape experiments, the NTP reaction mixture consisted of imaging buffer supplemented with all four NTPs. The final concentration of ATP in the observation chamber was 200 μM, while that of UTP, GTP and CTP was 100 μM. The CRIFF autofocusing system (ASI) was used during data acquisition to ensure focal stability.

The frame rates used for experiments are given in the relevant figure legends. For 20-ms frame rate experiments, excitation powers of 8 mW (532 nm laser) and 4 mW (635 nm laser) were used. For 200-ms frame rate experiments, excitation powers of 0.5 mW (532 nm laser) and 0.15 mW (635 nm laser) were used. All experiments were performed at 21°C.

Data analysis and visualisation. Fluorescence intensities were extracted from EMCCD images using *twoTone* software (Holden et al., 2010). The uncorrected FRET efficiency (E*) was then calculated as described (Pinkney et al., 2012). We manually inspected intensity time trajectories and selected molecules for analysis according to the following criteria: 1) bleached in a single step; 2) circular PSF across DD, DA, and AA channels; 3) no donor photoblinking (although fluctuations were permitted); 4) no acceptor photoblinking, although we allowed for different ATTO647N states (Ha and Tinnefeld, 2012); 5) fluorescence intensities being within a limited range; 6) no defocusing; and 7) no nearby molecules as measured using a nearest-neighbour criterion.

We manually inspected the intensity time trajectories of molecules in abortive initiation experiments and classified them into groups. The baseline state was assigned to RP_O and any increase in FRET that surpassed E*=0.3 was assigned to an initial transcribing complex with the transcription bubble in a scrunched conformation. We refer to these FRET states as scrunched FRET states. Time trajectories showing a stable scrunched state signal that lasted for >120 s were classified as stably scrunched. Time trajectories showing cyclical increases and decreases in FRET between the baseline and the scrunched FRET states were classified as cycling events in which RNA is synthesized during the increase in FRET, and released as abortive RNA during the decrease in FRET. Molecules showing a stepped increase in FRET, where the step lasted for >4 frames were classified as pausing molecules. Molecules showing a decrease in FRET from a maximally scrunched FRET state (excursion state) to a scrunched state above the baseline that lasted for >4 frames were classified as backtracked molecules. Molecules that bleached within 120 s of the addition of the NTP reaction mixture were excluded from analysis, since they could not be classified as either stably scrunched or cycling. The maximum FRET value reached in an $RP_{ITC\leq N}$ experiment was assigned as the FRET value corresponding to RNA molecules N-nt in length (see *Results*). For promoter-escape experiments, we identified specific patterns of signal changes that we assigned to promoter-escape events (see *Results*). For both abortive initiation and promoter escape experiments, molecules exhibiting confounding photophysical fluctuations were excluded from analysis.

HMM Analysis of cycling smFRET time trajectories. HMM analysis was performed on time trajectories displaying dynamic FRET fluctuations using custom-written MATLAB software (Le Reste et al., 2012; Uphoff et al., 2011). For abortive-initiation experiments, each time trajectory was fitted with 1 to 3 states and the best model (number of states) was selected automatically using maximum evidence criteria (Bronson et al., 2009; Uphoff et al., 2011). The FRET efficiency states above the baseline were classified as scrunched states. The dwell times of the scrunched states were extracted and used to generate dwell time distributions, which were fitted with an exponential decay curve. The mean scrunched-state dwell times were extracted from the fits.

HMM analysis was also performed to extract the mean dwell times of the paused and excursion states. These states were categorized according to their FRET efficiency and the states that preceded and followed them. FRET states that were preceded by the baseline and were followed by a higher FRET state were assigned as paused states. FRET states that were preceded by the paused state and were followed by backtracked or baseline states were assigned as excursion states. The dwell times of the states were used to generate dwell time distributions, which were fitted with exponential decay curves. The mean paused state dwell times (τ_{pause}) and the mean excursion dwell times (τ_{excurs}) were extracted from the fits.

Accessible volume (AV) modelling. An *E. coli* RP_O model was used to model the positions of the donor and acceptor dyes during the initial transcription reaction. The model was built using structural alignments to introduce the *E.coli* holoenzyme (PDB 4IGC, Murakami, 2013) and downstream DNA (using the *Tth* RNAP complex with downstream fork-junction DNA; PDB 4G7H, Zhang et al., 2012) in the *Taq* RP_o model (Murakami et al., 2002); downstream DNA beyond position +21 (and up to +35) was further extended as B-DNA. The AV of the donor and acceptor dyes attached to the DNA via a flexible linker were calculated according to (Muschielok et al., 2008). Here, the sterically accessible positions of the dye were obtained from lattice calculations performed on a flexible linker (length=14 Å, width=4.5 Å) attached to the C5 atom of the dT base with the dye modelled as a sphere (with a radius of 7 Å). The accessible volumes were approximated by a 3D Gaussian, the centre of which was approximated as the average position of the dyes. Visualisations and distance measurements between the donor and acceptor average dye positions were performed in PYMOL.

In RP_O, the donor dye was modelled attached to position -15, and the acceptor dye attached to position +20. For each successive nucleotide incorporation step that results in DNA scrunching, we modelled the acceptor dye attached to a position 1-bp upstream of the previous attachment position. Therefore, modelling the acceptor dye attached to +19, +18, +17, +16, and +15 approximated the average positions of the acceptor dye in RP_{ITC3}, RP_{ITC4}, RP_{ITC5}, RP_{ITC6}, and RP_{ITC7}, respectively. The donor dye was modelled still attached to position -15. In all AV models, the donor dye was modelled attached to the non-template strand, and the acceptor dye was modelled attached to the template strand.

REFERENCES

Bronson, J.E., Fei, J., Hofman, J.M., Gonzalez, R.L., and Wiggins, C.H. (2009). Learning rates and states from biophysical time series: a Bayesian approach to model selection and single-molecule FRET data. Biophys. J. *97*, 3196–3205.

Ha, T., and Tinnefeld, P. (2012). Photophysics of fluorescent probes for single-molecule biophysics and super-resolution imaging. Annu. Rev. Phys. Chem. *63*, 595–617.

Morichaud, Z., Chaloin, L., and Brodolin, K. (2016). Regions 1.2 and 3.2 of the RNA Polymerase σ Subunit Promote DNA Melting and Attenuate Action of the Antibiotic Lipiarmycin. J. Mol. Biol. *428*, 463–476.

Murakami, K.S., Masuda, S., Campbell, E.A., Muzzin, O., and Darst, S.A. (2002). Structural basis of transcription initiation: an RNA polymerase holoenzyme-DNA complex. Science *296*, 1285–1290.

Murakami, K.S. (2013) X-ray crystal structure of *Escherichia coli* RNA polymerase σ70 holoenzyme. J.Biol.Chem. *288*, 9126–34.

Muschielok, A., Andrecka, J., Jawhari, A., Brückner, F., Cramer, P., and Michaelis, J. (2008). A nano-positioning system for macromolecular structural analysis. Nat. Methods *5*, 965–971.

Tupin, A., Gualtieri, M., Leonetti, J.-P., and Brodolin, K. (2010). The transcription inhibitor lipiarmycin blocks DNA fitting into the RNA polymerase catalytic site. EMBO J. *29*, 2527–2537.

Uphoff, S., Gryte, K., Evans, G., and Kapanidis, A.N. (2011). Improved temporal resolution and linked hidden Markov modeling for switchable single-molecule FRET. Chemphyschem *12*, 571–579.

Zhang, Y., Feng, Y., Chatterjee, S., Tuske, S., Ho, M.X., Arnold, E., and Ebright, R.H. (2012). Structural basis of transcription initiation. Science *338*, 1076–80.

FIGURE S1 (related to Figure 1)

A

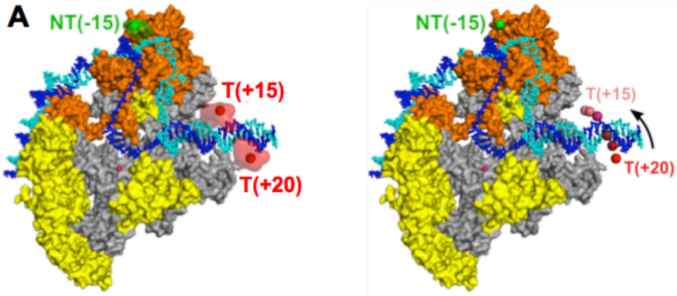

Acceptor position in RP$_o$	Equivalent RP$_{ITC}$ state	Distance R$_{model}$ (Å)	E$_{model}$	E$_{exp}$ (E*)	E$_{exp, corr}$	Distance R$_{exp}$ (Å)
+20	ITC2	107	0.04	0.22±0.01	0.04	105.3
+19	ITC3	102	0.05			
+18	ITC4	92	0.09	0.32±0.01	0.13	85.1
+17	ITC5	82	0.16	0.36±0.01	0.21	77.3
+16	ITC6	76	0.23	(0.37±0.01)	(0.22)	76.6
+15	ITC7	73	0.27	(0.45)	(0.31)	70.8

B

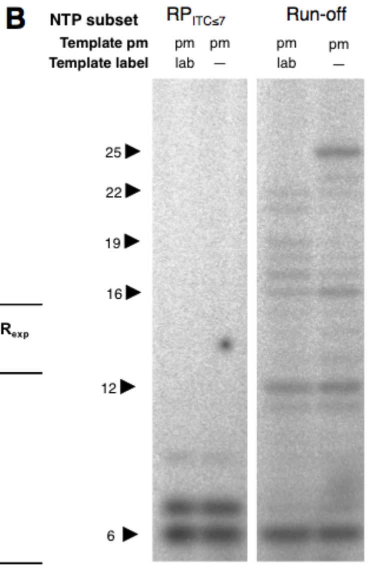

C

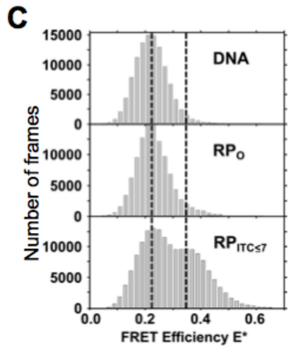

D

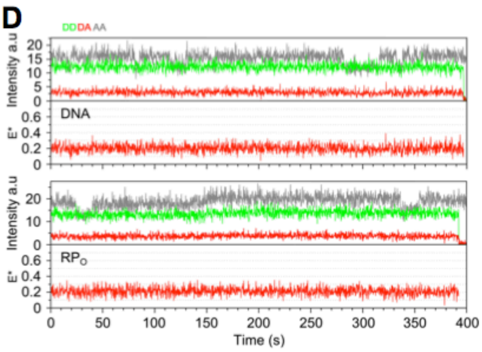

Figure S1. A single-molecule FRET assay for monitoring initial transcription in real-time.

(A) Fluorophore movement during initial transcription. Left, accessible-volume model of the donor fluorophore attached to -15 in RP$_O$ and in RP$_{ITC7}$, and the acceptor dye attached to +20 in RP$_O$ and +15 in RP$_{ITC7}$. The accessible volumes and mean positions of the fluorophores are shown. In both positions, the acceptor dye does not clash with protein. Right, accessible-volume model of RNAP during initial transcription. The acceptor dye is attached at position +20 and moves towards the donor during initial transcription. The final position in the model is +15, which corresponds to RP$_{ITC7}$. The distances between the donor fluorophore at position -15 and the acceptor fluorophore at various positions on the downstream DNA (as determined using accessible-volume modeling) is shown on the Table in the lower panel, along with the expected FRET efficiencies E$_{model}$. For a comparison, the measured uncorrected FRET efficiencies E$_{exp}$ are shown, along with the corrected FRET efficiencies E$_{exp, corr}$ and the corresponding experimental distances R$_{exp}$.; the first decimal point is shown in order to emphasize the expected *change* in distance at the point of the FRET dynamic range. For the assignment of FRET states to RP$_{ITC}$ complexes, see main text.

(B) *In vitro* transcription as a functional test for the labelled, pre-melted promoter fragment. [α^{32}P]GTP was used in both panels. The initial transcribed sequence is 5'-AAUUGUG-3'. Left panel, testing for any effects of the donor and acceptor fluorophores on the abortive profile of RNAP on the pre-melted lac*CONS* promoter. Right panel, testing for any effects of the donor and acceptor fluorophores on promoter escape and early elongation by RNAP on the pre-melted *lac*CONS promoter.

(C) Immobilized free promoter DNA and RP$_{ITC2}$ show no fluctuations to higher FRET values. Top panel: stacked E* histograms of surface-immobilized DNA (top). Middle panel: RPo plus ApA (RP$_{ITC2}$). Bottom panel: RP$_{ITC≤7}$. Each histogram was constructed using data from six fields-of-view. The NTP reaction mixture was added before starting data acquisition. Frame rate is 20 ms.

(D) Example time-traces of DNA (top) and RP$_o$ plus ApA (bottom). FRET remained constant at E*~0.23 for many minutes. ATTO647N intensity state changes can be seen in the AA channel. Frame time: 200 ms.

FIGURE S2 (related to Figure 2)

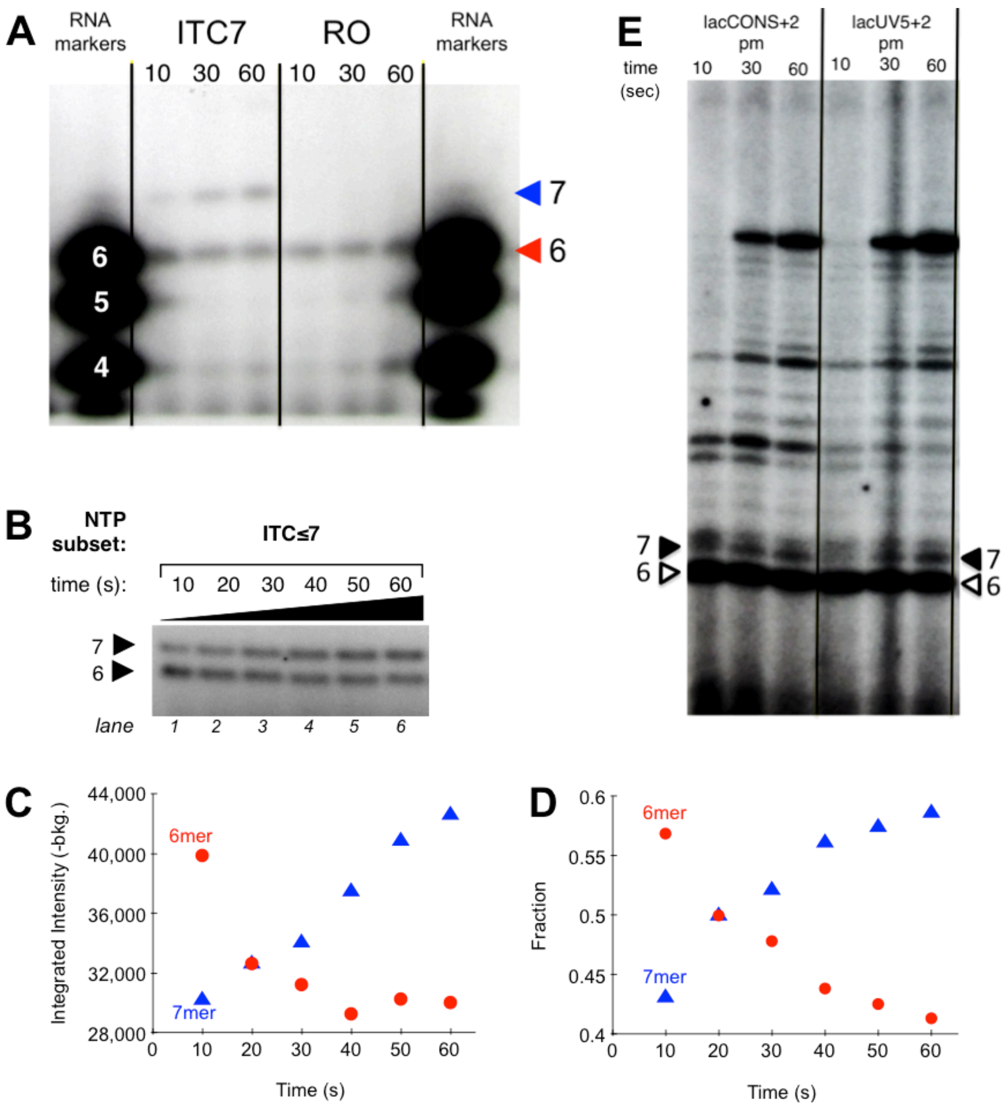

Figure S2. Quantitation of *in vitro* transcription reaction time-points.
(A) Length assignment of short RNA products. *In vitro* transcription gels of RNA products produced by transcription complexes able to synthesize up a 7-nt RNA ("ITC7") and up to a run-off product ("RO"), compared with RNA markers. In both cases, the 5'-end of the RNA is phosphorylated; in the case of transcription, this is achieved by using pApA as a dinucleotide primer. RNA markers are phosphorylated, and carry the exact sequence of the first *lac*CONS (or *lac*UV5) transcripts: the 4-mer is 5'-pAAUU-3', the 5-mer is 5'-pAAUUG-3' and the 6-mer is 5'-pAAUUGU-3'. The mobility comparison clearly shows that the main band in ITC7 as well as the run-off reaction is the 6-mer. Note that although the gel is continuous, black lines have been added for annotation and clarity.

(B) *In vitro* transcription reactions under $RP_{ITC\leq7}$ conditions (for details, see Fig. 2C) reveal a pause event after synthesis of a 6-mer RNA product. Transcription products were detected by phosphorimaging (Fujifilm).

(C-D) RNA-band quantitation using ImageJ (panel C); the amount of the 6- vs. 7-mer product was normalized to the sum of 6- and 7-mer band intensities at each time-point (panel D).

(E) Pausing on *lac*UV5 promoter. *In vitro* transcription gels under transcription runoff conditions (using all NTPs) showing the presence of a 6-mer pause both on *lac*CONS+2 and *lac*UV5 promoters. The promoters differ on the strength of their -35 element (lacCONS+2: TTGACA, matching the -35 consensus; *lac*UV5+2: TTTACA, having a 5 out of 6 match to the consensus) and the degree of match to the consensus length for the -10/-35 spacer (*lac*CONS+2, 17-bp, matching the consensus; *lac*UV5, 18-bp). The profile of short products (up to 10 nt) which reports on pausing after synthesis of a 6-mer, is identical for both promoters. Note that although the gel is continuous, black lines have been added for annotation and clarity.

FIGURE S3 (related to Figure 4)

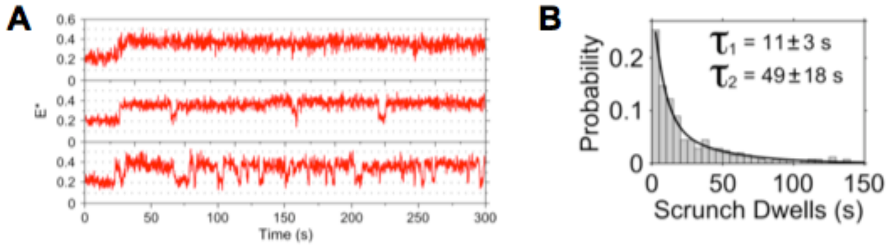

Figure S3. The main initial transcription behaviors in $RP_{ITC\leq7}$ are independent of the bubble mismatch. Frame time: 200 ms.

(A) Example time-traces of stable scrunched (top), and abortive cycling (middle and bottom) immobilized $RP_{ITC\leq7}$ complexes formed using fully complementary DNA (dsDNA).

(B) Dwell-time distribution for dsDNA using abortive cycling data (*N*=250). The scrunched state lifetimes result from the fit of the distribution to a bi-exponential decay; the amplitudes of the short and long lifetimes are ~70% and ~30%, respectively.

FIGURE S4 (related to Figure 6)

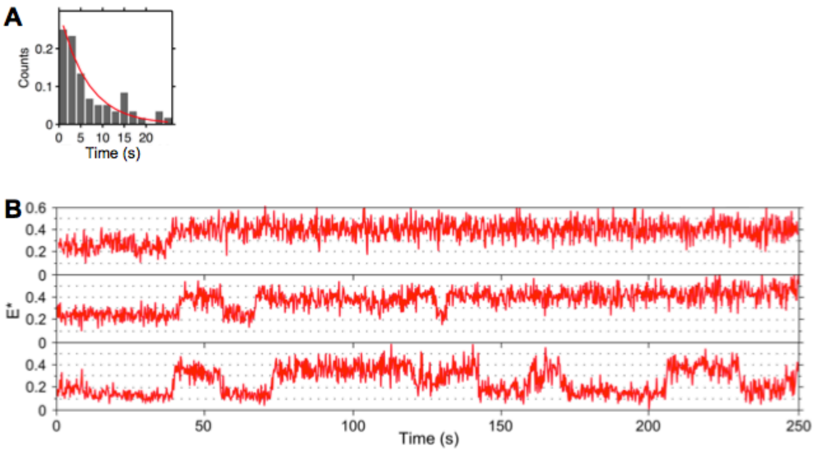

Figure S4. Analysis of complexes provided with all four NTPs to allow promoter escape.

(A) Dwell-time histogram for the escape pause; the average dwell time is 7.7 s (*N*=62).

(B) Example time-traces of transcription complexes that, despite being provided the full set of NTPs for promoter escape, show abortive-cycling behaviors similar to those seen for $RP_{ITC\leq7}$ complexes.
