# Supplementary material for: RNA Polymerase Pausing during Initial Transcription
Source: Mol Cell. 2016 Sep 15;63(6):939–50. doi: 10.1016/j.molcel.2016.08.011 (PMC5031556; doi:10.1016/j.molcel.2016.08.011)
Supplement: Document S2. Article plus Supplemental Information [file mmc2.pdf]

Article

Molecular Cell

RNA Polymerase Pausing during Initial Transcription

Graphical Abstract

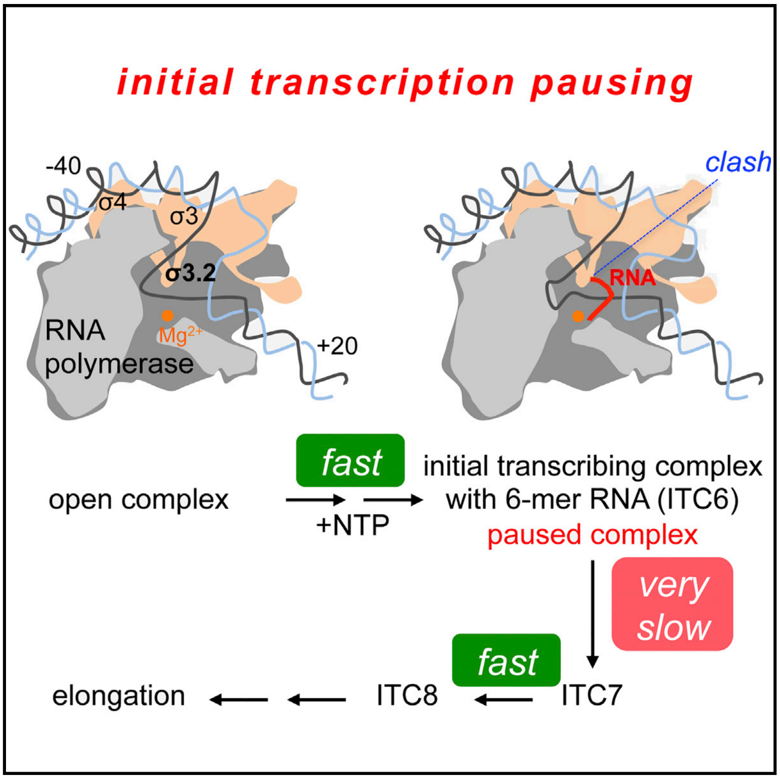

Authors

Diego Duchi, David L.V. Bauer, Laurent Fernandez, ..., Zakia Morichaud, Konstantin Brodolin, Achillefs N. Kapanidis

Correspondence

kapanidis@physics.ox.ac.uk

In Brief

Initial transcription involves synthesis of short RNAs that are either released or extended to allow promoter escape; such mechanisms are unclear due to heterogeneity. Duchi et al. used single-molecule fluorescence to visualize transcription in real time and discovered a long pause after synthesis of a 6-mer RNA.

Highlights

- *E. coli* RNA polymerase pauses during initial transcription at *lac* promoters
- Initiation pausing lasts for ~20 s and occurs at the transition from 6- to 7-nt RNA
- Region 3.2 of σ^{70} is the main protein element controlling pausing
- Pausing is likely to be controlled further by a complex set of determinants

CrossMark

Duchi et al., 2016, Molecular Cell *63*, 939–950
September 15, 2016 © 2016 The Author(s). Published by Elsevier Inc.
http://dx.doi.org/10.1016/j.molcel.2016.08.011

CellPress

CellPress

RNA Polymerase Pausing during Initial Transcription

Diego Duchi,[1] David L.V. Bauer,[1] Laurent Fernandez,[1] Geraint Evans,[1] Nicole Robb,[1] Ling Chin Hwang,[1] Kristofer Gryte,[1] Alexandra Tomescu,[1] Pawel Zawadzki,[1] Zakia Morichaud,[2] Konstantin Brodolin,[2] and Achillefs N. Kapanidis[1,3,*]
[1]Biological Physics Research Group, Clarendon Laboratory, Department of Physics, University of Oxford, Oxford OX1 3PU, UK
[2]CNRS FRE 3689, Centre d'études d'agents Pathogénes et Biotechnologies pour la Santé (CPBS), 1919 route de Mende, 34293 Montpellier, France
[3]Lead Contact
*Correspondence: kapanidis@physics.ox.ac.uk
http://dx.doi.org/10.1016/j.molcel.2016.08.011

SUMMARY

In bacteria, RNA polymerase (RNAP) initiates transcription by synthesizing short transcripts that are either released or extended to allow RNAP to escape from the promoter. The mechanism of initial transcription is unclear due to the presence of transient intermediates and molecular heterogeneity. Here, we studied initial transcription on a *lac* promoter using single-molecule fluorescence observations of DNA scrunching on immobilized transcription complexes. Our work revealed a long pause ("initiation pause," ~20 s) after synthesis of a 6-mer RNA; such pauses can serve as regulatory checkpoints. Region sigma 3.2, which contains a loop blocking the RNA exit channel, was a major pausing determinant. We also obtained evidence for RNA backtracking during abortive initial transcription and for additional pausing prior to escape. We summarized our work in a model for initial transcription, in which pausing is controlled by a complex set of determinants that modulate the transition from a 6- to a 7-nt RNA.

INTRODUCTION

Transcription initiation is the most highly regulated step in gene expression. In bacteria, RNA polymerase (RNAP) binds to promoter DNA and unwinds ~14 bp around the transcription start site to form a transcription bubble, with the unwound template (T) strand moving into the RNAP active center cleft. This conformational change leads to the formation of the RNAP-promoter open complex, RP_o (Murakami and Darst, 2003; Saecker et al., 2011), which then engages in de novo RNA synthesis via productive or abortive pathways (Carpousis and Gralla, 1980; Hsu, 2002). In the productive pathway, RNAP synthesizes RNA within an RNAP-promoter initial transcribing complex (ITC); when the nascent RNA becomes 9- to 11-nt long, RNAP escapes from the promoter and enters elongation (Mukhopadhyay et al., 2001; Murakami and Darst, 2003). In the abortive pathway (also known as abortive initiation), RNAP synthesizes short RNAs, but does not escape from the promoter; instead, RNAP releases short RNAs, reverts back to RP_o, and re-initiates RNA synthesis (Carpousis and Gralla, 1980; Gralla et al., 1980). The balance between productive and abortive pathways depends on the promoter and initial transcribed sequences (Hsu, 2009).

Despite this progress, which has been aided by structures of ITCs (Basu et al., 2014; Zuo and Steitz, 2015), our understanding of initial transcription is limited, in part due to the heterogeneity and dynamics of the complexes involved (Hsu, 2002, 2009; Kubori and Shimamoto, 1996). Such issues are addressable by single-molecule studies, which can also examine reactions in real time without synchronization. In early work, we used single-molecule Förster resonance energy transfer (smFRET) confocal microscopy (Kapanidis et al., 2004, 2005a) to monitor multiple distances within diffusing transcription complexes and showed that initial transcription proceeds via a DNA-scrunching mechanism (Kapanidis et al., 2006), during which RNAP unwinds and pulls downstream DNA into its active site cleft. DNA nanomanipulation work also showed that scrunching occurs in initial transcription and is obligatory for escape (Revyakin et al., 2006).

However, the confocal smFRET study offered only short (~1 ms) structural snapshots of transcription complexes. An early smFRET work on immobilized complexes (Margeat et al., 2006) was also limited by low temporal resolution, short observations, and photophysical fluctuations. In contrast, the DNA nanomanipulation work offered long observations, but did not identify kinetically stable intermediates. As a result, the mechanism, kinetics, and regulation of initial transcription have remained unclear. There is also a need to evaluate the role of σ^{70} region 3.2 (σ3.2) in initial transcription, since it is a major determinant of abortive initiation (Murakami et al., 2002).

Here, we use an optimized smFRET strategy to monitor de novo RNA synthesis in real time by monitoring DNA scrunching, which occurs concomitantly with each nucleotide incorporation in initial transcription (Figure 1A). Surprisingly, we observe highly stable scrunched states and extensive pausing during initial transcription, with region σ3.2 being a major pausing determinant. We also obtained evidence for RNA backtracking during abortive initial transcription, and for additional pausing prior to escape. Our results were summarized in a model for initial transcription, in which pausing is controlled by a complex set of determinants that modulate the transition from a 6- to a 7-nt RNA.

This is an open access article under the CC BY license (http://creativecommons.org/licenses/by/4.0/).

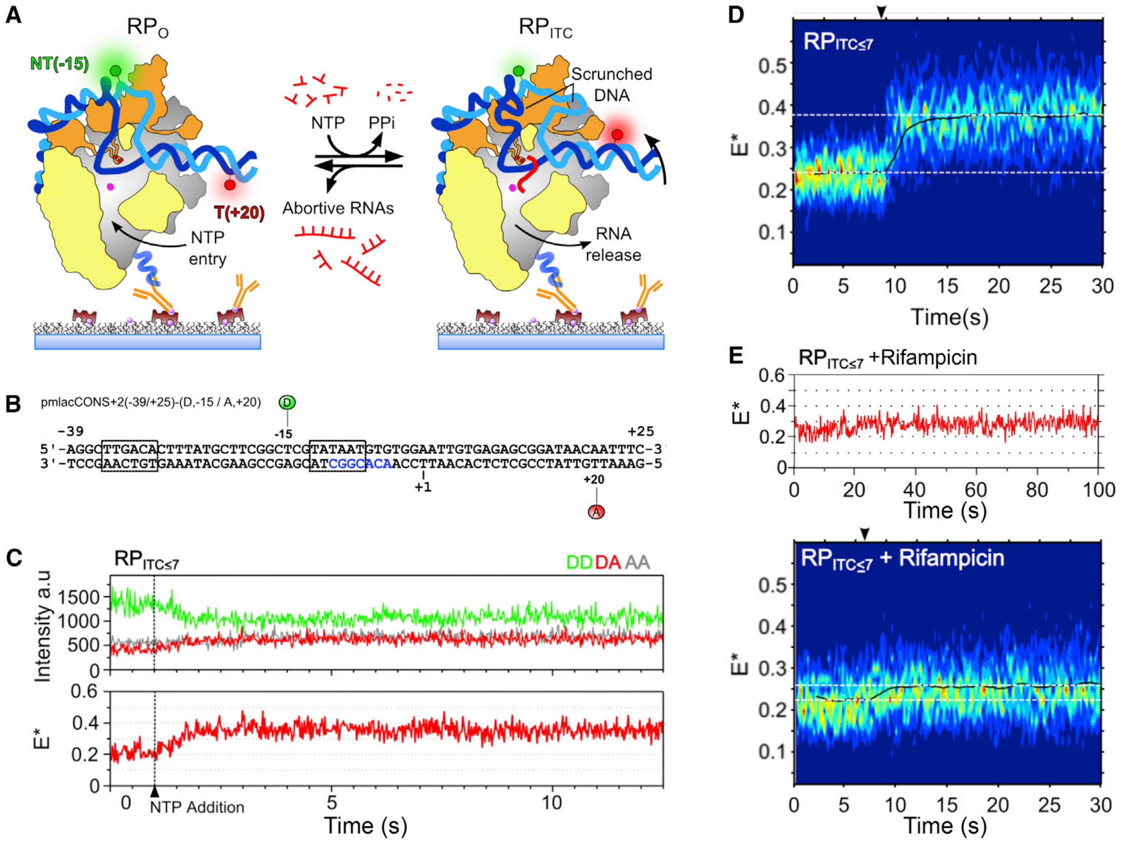

Figure 1. A Single-Molecule FRET Assay for Real-Time Initial Transcription

(A) Schematic of assay. Left, RP_o; right, initial transcribing complex (ITC). Donor is in green; acceptor in red; σ^{70} in orange; RNAP in gray, except for the β subunit (omitted for clarity) and regions protruding from the cut-away plane (in yellow); template strand in blue; non-template strand in teal; nascent RNA in red; and RNAP active site in pink. The penta-His antibody anchors RP_o to the surface. The initial FRET efficiency is low; upon NTP addition, scrunching moves the acceptor closer to the donor, increasing FRET efficiency.

(B) *lac*CONS DNA fragment for FRET assay; the $-10/-4$ pre-melted region is in blue.

(C) Time trace showing an increase to E*~0.37 upon adding 80 μM UTP and GTP to form $RP_{ITC\leq7}$. The NTP addition point is marked with a dashed line. Frame time: 20 ms. DD trace (green trace, top), donor emission upon donor excitation; DA trace (red trace, top), acceptor emission upon donor excitation; AA trace (gray trace, top), acceptor emission upon acceptor excitation. DD and DA are used for calculating apparent FRET efficiency E*.

(D) Transcription heatmaps (n = 45) showing activity upon NTP addition to form $RP_{ITC\leq7}$. NTP addition is marked by an arrowhead. Blue to red colors represent an increasing number of events. Black line, time trace of average E* of all traces; white dotted lines, E* for RP_o baseline (at E*~0.24) and RP_{ITC} plateau (at E*~0.37). Frame time: 200 ms.

(E) Time trace (top) and transcription heatmap (bottom, n = 37) for $RP_{ITC\leq7}$ in the presence of rifampicin.

See also Figure S1.

RESULTS

Real-Time Initial Transcription by Single RNAP Molecules

To study initial transcription in real time, we used smFRET to monitor DNA conformational changes within surface-immobilized transcription complexes. We used DNAs based on a derivative of *lac* promoter (*lac*CONS), a promoter rate-limited in initial transcription (Carpousis and Gralla, 1980; Gralla et al., 1980). We monitored FRET between fluorophores flanking the transcription bubble (Kapanidis et al., 2006; Margeat et al., 2006; Robb et al., 2013); the donor was placed in the $-10/-35$ spacer DNA (at position -15 of the non-template DNA) and the acceptor on the DNA downstream of the bubble (at position +20 of the template DNA; Figure 1A). The initial FRET efficiency for this pair in RP_o was expected to be low: as RNAP synthesizes short RNAs (2- to 7-mer), the downstream DNA flanking the acceptor should rotate and approach the donor, leading to a FRET increase (Figure 1A; for the expected donor-acceptor distances and FRET efficiencies, see Figure S1A, available online). To maximize the yield of active immobilized complexes, we used a pre-melted version of *lac* DNA (pmDNA; Figure 1B); the FRET pair on the DNA did not affect either the *lac* abortive profile or the ability of RNAP to escape (Figure S1B).

To measure the FRET efficiency in RP_o complexes, we anchored them to a polyethylene glycol (PEG)-coated surface and imaged them via total internal reflection fluorescence (TIRF) microscopy (Figure 1A). Immobilized RP_o in the presence

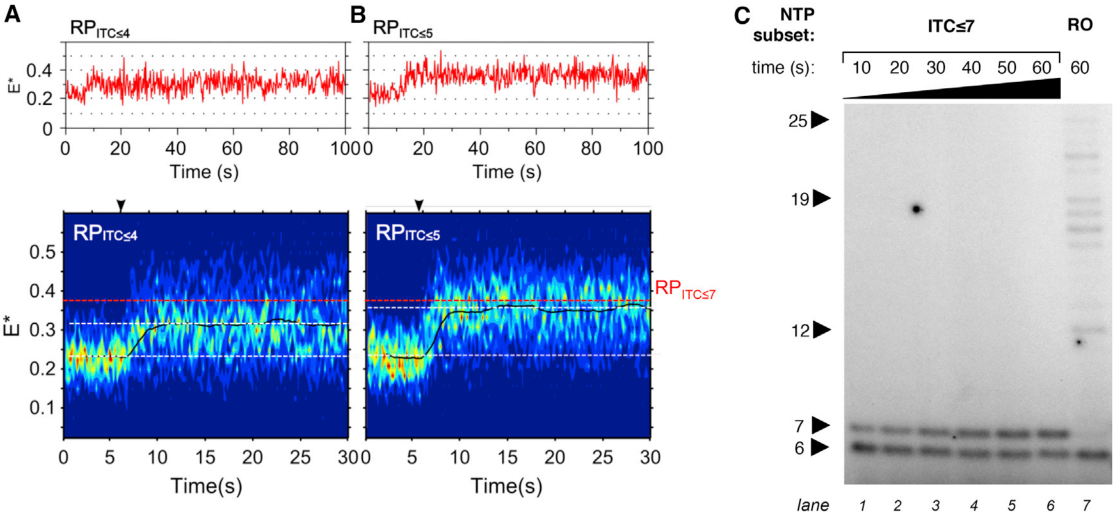

Figure 2. A Pause during RNA Extension from 6 to 7 nt in Length

(A and B) FRET time traces (top) and heatmaps (bottom) for all active $RP_{ITC\leq4}$ (n = 45; A) and $RP_{ITC\leq5}$ (n = 53; B) complexes. Style as in Figure 1. The dotted red line at E*~0.37 marks the high-FRET plateau for $RP_{ITC\leq7}$ (Figure 1D).

(C) Transcription activity for $RP_{ITC\leq7}$ and run-off products on *lac*CONS. Lanes 1–6 follow RNAs made under $RP_{ITC\leq7}$ conditions (RP_o + 500 μM ApA, 80 μM UTP, and 80 μM GTP) over 60 s. Lane 7 represents the run-off reaction (RP_o + 500 μM ApA, and 80 μM of all NTPs). The RNA length was assigned by comparison with length standards with sequences identical to the short RNAs produced on *lac*CONS; see Figure S2A. The gel shows no accumulation of RNAs shorter than 6 nt under our conditions; we note that 3- to 4-mers are also produced (see Figure 5F), but are not recovered well by the precipitation step prior to gel loading. See also Figure S2.

of dinucleotide ApA (RP_{ITC2}) formed the same stable FRET state as DNA alone (FRET efficiency [E*]~0.22; Figure S1C, top and middle panels) and did not reach higher FRET states (Figure S1D).

To observe initial transcription in real time, we provided immobilized RP_o complexes with subsets of nucleotides, trapping RNAP in iterative abortive synthesis and preventing promoter escape (Carpousis and Gralla, 1980; Kapanidis et al., 2006). Specifically, we added ApA, UTP, and GTP to form complexes limited to synthesis of RNA of up to 7 nt in length ($RP_{ITC\leq7}$; with the longest RNA being 5′-AAUUGUG-3′). Addition of 80 μM UTP and GTP (at ~1 s; Figure 1C) indeed led to a gradual decrease in donor fluorescence and an anticorrelated increase in acceptor fluorescence (DD and DA traces; Figure 1C, top); these signals corresponded to a gradual FRET increase from the RP_o state (E*~0.2, initial segment of FRET trace; Figure 1C) to a higher FRET state (E*~0.37). After the initial increase (completed in ~1 s), the FRET signal was stable, indicating that the E*~0.37 state is stable for >10 s.

To study all active complexes on a single field of view (n ~ 50), we superimposed their FRET traces on a "transcription heat map" (Figure 1D). The map showed that the large majority of molecules display the same behavior of gradual increase (in 1–2 s) from RP_o to a higher FRET state (E* = 0.37 ± 0.01, mean ± SEM), which was occupied for >20 s. To test whether the increase was due to transcription, we performed controls wherein we added UTP and GTP to immobilized RP_{ITC2} in the presence of rifampicin, an inhibitor that blocks synthesis of RNA of >3 nt in length (Campbell et al., 2001; McClure and Cech, 1978). Our results showed only a small change (~0.04) in the presence of rifampicin upon UTP/GTP addition (Figure 1E), likely due to the RNAP being able to extend ApA to a 3-nt RNA.

RNAP Pauses after Synthesizing a 6-nt RNA

To monitor scrunching in different ITCs, we followed FRET during the first few nucleotide additions: we formed $RP_{ITC\leq4}$ and $RP_{ITC\leq5}$ complexes, generated their heatmaps, and compared them to $RP_{ITC\leq7}$ with regards to the magnitude of FRET increase and the stability of the highest FRET state (Figure 2A). For $RP_{ITC\leq4}$, a plateau at E*~0.32 was reached in ~2 s after NTP addition (Figure 2A, bottom); the range of FRET values at the plateau was wider than for $RP_{ITC\leq7}$, mainly reflecting the lower stability of shorter RNA within ITCs. For $RP_{ITC\leq5}$, a higher plateau (E*~0.36) was reached in ~2 s after NTP addition (Figure 2B, bottom); the range of FRET values at the plateau was as for $RP_{ITC\leq7}$.. To compare the FRET-based distance changes to structural model predictions, we calculated the corrected FRET efficiencies for the stable scrunched states and their corresponding distances (Figure S1A); while the observed distance decrease upon going from the stable scrunched state of $RP_{ITC\leq4}$ to that of $RP_{ITC\leq5}$ was similar to the model prediction (ΔR_{model} ~10 Å; ΔR_{exp} ~8 Å), the distance decrease for the transition from $RP_{ITC\leq5}$ to $RP_{ITC\leq7}$ was much smaller than expected (ΔR_{model} ~9 Å; ΔR_{exp} ~1 Å), raising the possibility that the main abortive RNAs in $RP_{ITC\leq7}$ were shorter than a 7-mer.

To obtain the distribution of short transcripts at our promoter for $RP_{ITC\leq7}$, we performed in vitro transcription (Figure 2C; for gel band assignment, see Figure S2A). The results showed that $RP_{ITC\leq7}$ synthesized a substantial fraction of 6-nt RNA (5′-AAUUGU-3′), an RNA one nucleotide shorter than expected for this complex. At short incubations (10–20 s, similar to the timescale for the FRET measurements), the 6-nt RNA was the main product and was slowly extended ($t_{1/2}$ ~20 s) to a 7-mer (5′-AAUUGUG-3′); the 7-mer became the main product in 60 s

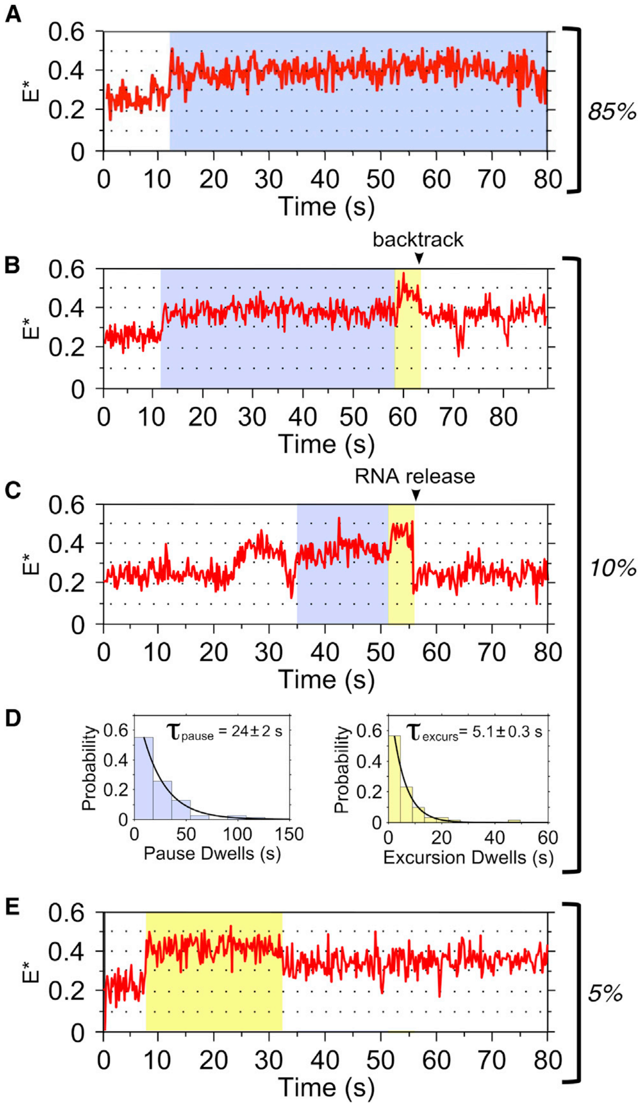

Figure 3. Single-Molecule Transcription by $RP_{ITC\leq7}$ Complexes
Frame time: 200 ms.
(A) Time trace showing an increase to a stable E*∼0.37 state.
(B) Time trace showing pausing at E*∼0.37 (highlighted blue), followed by an excursion to the E*∼0.45 state (highlighted yellow). The return to the stable E*∼0.37 is assigned to RNA backtracking.
(C) Time trace showing pausing, followed by an excursion to E*∼0.45 (as in B), followed by a return to the RP_o baseline (assigned to RNA release).
(D) Dwell-time histograms and exponential fits for the paused state (left; n = 84) and the E*∼0.45 state (right; n = 60).
(E) Time trace showing no pausing before reaching E*∼0.45, followed by a return (highlighted yellow) to a stable E*∼0.37 state.

(Figures S2B–S2D). This behavior is the hallmark of transcriptional pausing. Importantly, the 6-nt RNA was also present for complexes supplied with all NTPs (run-off; Figure 2C, lane 7), showing that the paused complex at 6-nt RNA was an on-pathway intermediate. In contrast, the 7-nt RNA was almost absent in the run-off reaction, showing that extension beyond a 7-mer was efficient, and that there was no significant pausing after synthesis of a 7-mer; we obtained identical results on a *lac*UV5 promoter (which differs from *lac*CONS by not having a consensus −35 and consensus −10/−35 spacer; Figure S3). The prevalence of a 6-mer RNA in ITCs capable of synthesizing a 7-mer was consistent with studies on *lac*UV5 (Brodolin et al., 2004; Carpousis and Gralla, 1980).

RNAP Pausing during Initial Transcription by Single ITCs

To further study the FRET states in $RP_{ITC\leq7}$, we examined individual traces. As expected, ∼85% of all traces (221 of 260) showed complexes reaching the long-lived state of E*∼0.37 (Figure 3A). Based on our in vitro transcription results (where the 6-mer accumulates before converting to a 7-mer), we assigned the E*∼0.37 state to a complex with an RNA of 6 nt in length (i.e., RP_{ITC6}). We also saw that in ∼15% of the traces (n = 39), an E*∼0.45 state is reached, which we assigned to RP_{ITC7}, a complex containing a 7-mer RNA (the longest RNA synthesized with the nucleotide subset used); in ∼65% of these traces (n = 25), the E*∼0.45 state was reached after a pause at E*∼0.37 for several seconds (Figures 3B and 3C), while in the rest of the traces, the E*∼0.45 state was reached without apparent pausing (Figure 3E). These results, along with our in vitro transcription, suggest that RNAP enters a long-lived paused state after synthesizing a 6-nt RNA, which is then slowly extended to a 7-nt RNA.

To study the pausing kinetics, we plotted the pause-time distribution for molecules that occupied the E*∼0.37 state before the E*∼0.45 state. The distribution fitted well to a single-exponential decay (indicating a single rate-limiting step) with a duration of 24 ± 2 s (Figure 3D, left); this long lifetime suggests that the pause could be rate-limiting for promoter escape.

Once the E*∼0.45 state was reached, the complex either returned to the E*∼0.37 state (Figures 3B and 3E) or the RP_o baseline (Figure 3C). On average, the lifetime of the E*∼0.45 state was 5.1 ± 0.3 s (Figure 3D, right). Since RNAP can form 7-mers (Figure 2C), the return to the E*∼0.37 was likely due to RNA backtracking in RP_{ITC7} to the translocational register seen for the 6-mer RNA (see Discussion). Further, the return to the RP_o baseline, frequently followed by additional cycling to higher FRET states, is consistent with abortive RNA release.

Scrunched Complexes Are Stable after Synthesis of a 6-nt RNA

We then examined the stability of $RP_{ITC\leq7}$ complexes occupying the E*∼0.37 state (RP_{ITC6}) by analyzing complexes retaining their FRET pair for >10 min (Figure 4). About 45% of the complexes adopted a single E*∼0.37 state for >120 s (“stably scrunched complexes”; Figure 4A, top). The rest adopted scrunched states for <120 s, followed by a return to the RP_o baseline and new rounds of RNA synthesis (“cycling complexes”; Figure 4A, middle and bottom).

To evaluate the stability of scrunched states in cycling complexes, we analyzed the distribution of dwell times in the scrunched state; the distribution exhibited bi-exponential decay kinetics with mean times of t_1∼8 s and t_2∼55 s (Figure 4B). The long-lived species is likely to be similar to the stably scrunched complexes. We obtained similar lifetimes for $RP_{ITC\leq7}$ complexes formed on a fully double-stranded

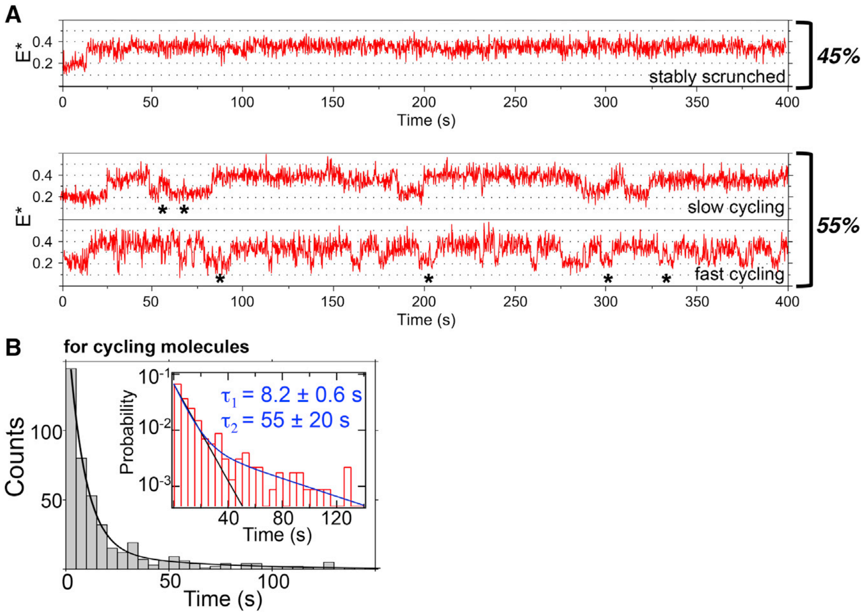

Figure 4. Single-Molecule Transcription by $RP_{ITC\leq7}$: Extended Observations

Frame time: 200 ms.

(A) Time traces of stable scrunched (top) and abortive cycling (middle and bottom) transcribing $RP_{ITC\leq7}$. Events that may show short (<5 nt) abortive RNAs being synthesized and released are marked with asterisks.

(B) Distribution of scrunched-state dwell times for cycling molecules (n = 445), shown in a linear and semi-log plot (inset). The distribution is fitted well by a short and a long lifetime (~85% and ~15% of the events, respectively); a single-exponential fit (black line in inset) fails to account for the population of long-lived dwells. Most short dwells come from fast cycling molecules.

See also Figure S3.

promoter DNA fragment (Figure S3), showing that the stability of scrunched complexes is unaffected by the mismatch in our pre-melted DNA.

Region σ3.2 Blocks RNA Extension beyond 6 nt

An explanation for the inability of most $RP_{ITC\leq7}$ complexes to rapidly synthesize a 7-nt RNA is the presence of structural elements that block motions for smooth progression from RP_{ITC6} to RP_{ITC7}; such elements may also destabilize the RP_{ITC7} state when reached, as suggested by the short dwell in the E*~0.45 state (Figures 3B, 3C, and 3E). A candidate for this role is σ region 3.2, a part of which forms an unstructured loop (also known as "σ finger") that partially occupies the RNA exit channel (Basu et al., 2014; Murakami, 2013; Zhang et al., 2012). Based on structural models, the 5′ end of RNA is expected to clash with σ3.2 when the RNA becomes 5- to 6-nt long (Figure 5A; Murakami et al., 2002; Zuo and Steitz, 2015). We thus tested whether σ3.2 affects RNA extension beyond a 6-mer; we also hypothesized that deleting σ3.2 would increase the yield of 7-nt RNAs produced by $RP_{ITC\leq7}$ and eliminate pauses en route to E*~0.45.

To test our hypotheses, we studied complexes formed using a mutant RNAP lacking part of σ3.2 (Δ3.2, lacking residues 513–519; Kulbachinskiy and Mustaev, 2006). The Δ3.2 mutant is expected to have a more accessible RNA exit channel and weaker interactions with the template strand. Indeed, Δ3.2 $RP_{ITC\leq7}$ complexes synthesized mainly a 7-nt RNA (Figure 5B, lane 1), as opposed to wild-type (WT) complexes, which synthesized similar amounts of a 6-nt and 7-nt RNA (Figure 5B, lane 4). Further, upon NTP addition that allows Δ3.2 RNAP to form an RNA of up to 11 nt in length (Figure 5B, lane 2), or a run-off product (a 25-nt RNA; Figure 5B, lane 3), the 6-nt RNA was greatly reduced (but not eliminated) relative to the amount for WT complexes, which synthesize a 6-mer as their main short transcript (Figure 5B, lanes 5 and 6). These results establish σ3.2 as a major pausing determinant after RNAP synthesizes a 6-mer on *lac*CONS. Notably, the fact that the 6-nt RNAs are not eliminated for Δ3.2 under all conditions ($RP_{ITC\leq7}$, RD_{e11}, and run-off) points to the presence of additional pausing determinants.

We performed similar comparisons using smFRET on $RP_{ITC\leq7}$ complexes and found major differences between the Δ3.2 and WT RNAP complexes. Heatmaps (Figure 5C) showed that Δ3.2 complexes sample higher FRET states more readily than WT (~17% ± 5% of Δ3.2 states show E*>0.45 versus ~6% ± 2% for WT; mean ± SD); this is despite the fact that Δ3.2 complexes with E*>0.3 are less stable and dissociate quickly, broadening the FRET distribution after NTP addition (E* full width at half maximum was ~0.34 for Δ3.2 and ~0.18 for WT; see also Figure 5C).

We then compared time traces of Δ3.2 and WT RNAP complexes (Figure 5D). First, Δ3.2 $RP_{ITC\leq7}$ complexes reached the E*~0.45 state more often than WT $RP_{ITC\leq7}$ complexes (72 of 219 molecules for Δ3.2, i.e., 33% ± 5% of all transitions versus 15% ± 5% for WT; mean ± SD). Second, the vast majority of Δ3.2 complexes that did reach the E*~0.45 state (90% of 47 molecules) did so without an apparent pause at E*~0.37 (Figure 5D); the same number for WT was only ~30%. Third, there was a large decrease in the fraction of stably scrunched molecules (15% ± 7% for Δ3.2 complexes versus 46% ± 5% for WT complexes). The scrunched states in the Δ3.2 $RP_{ITC\leq7}$ complex were also significantly less stable, as judged by the ~20% and ~50% decrease in the fast and slow scrunched-state lifetimes, respectively (Figure 5E). This observation suggests that σ3.2 acts not only as a barrier to the 6-mer extension, but also contributes to the stable attachment of the 6-mer within $RP_{ITC\leq7}$.

To further study the attachment of 6-mer to $RP_{ITC\leq7}$ complexes and its dependence on σ3.2, we performed in vitro transcription on bead-immobilized complexes and examined the profile of RNAs retained by the complexes after a 2 min wash (Figure 5F). Approximately 14% of the total 6-nt and 7-nt RNA is retained in the complex after the wash, which implies an average RNA retention lifetime of ~1 min. Identical experiments for Δ3.2 showed 3-fold lower retention for the 6-mer (and 2-fold

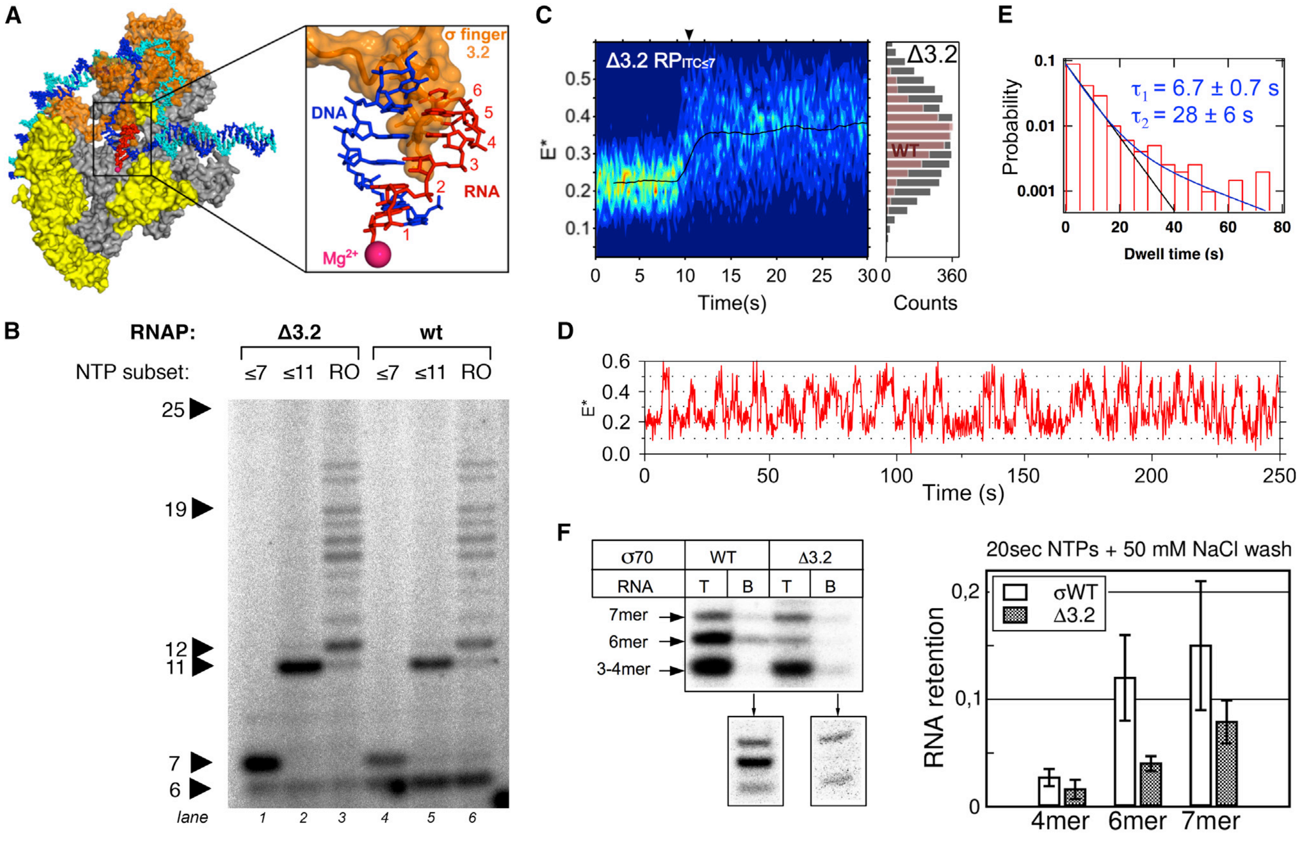

Figure 5. Region σ3.2 Is a Major Determinant of Initiation Pausing

(A) Structural model of an ITC highlighting the clash between nascent 6-nt RNA (in red) and σ3.2 (in orange). Colors as in Figure 1A.

(B) Comparison of transcription by Δ3.2 versus WT RNAP on *lac*CONS. Lanes 1–3: RNAs produced by Δ3.2 complexes able to synthesize up to 7-nt RNA (lane 1), up to 11-nt RNA (lane 2), and up to a run-off product (a 25-mer; lane 3). Lanes 4–6: RNAs produced by same mixtures as for lanes 1–3, but for WT RNAP.

(C) Heatmaps for Δ3.2 complexes in $RP_{ITC\leq7}$. Right-side histogram: collapse of all E* values in the high-FRET plateau (reached at ~12 s; gray bars). Frame time: 200 ms. The E* full width at half maximum was ~0.34 for Δ3.2, ~2-fold wider than for WT (0.18 ± 0.02, pink bars).

(D) Time trace where E*~0.45 is sampled frequently and without long pauses at E*~0.37.

(E) Dwell-time distributions of Δ3.2 scrunched states (n = 392).

(F) Retention of 6-nt and 7-nt RNAs in complexes due to σ3.2 presence. Reactions for $RP_{ITC<7}$ were run for 20 s at 37°C on bead-immobilized RP_o; reactions were stopped, and complexes were washed and incubated for ~2 min before gel loading. WT panel: using WT sigma and no washing ("T" lane), in vitro transcription yields 6-mers and 7-mers, as well as unresolved 3/4-mers. As for *lac*CONS, the 6-mer is more abundant than the 7-mer, consistent with pausing at 6-nt RNA. After washing and incubation ("B" lane), little 3/4-mer is retained; in contrast, there is much higher retention of 6-mer and 7-mer RNA. Lower inset: sample from lane B was run in a separate lane and overexposed. Δ3.2 panel: using Δ3.2 and no washing, in vitro transcription yields 6-mers, 7-mers, and unresolved 3/4-mers; as for *lac*CONS, with the 6-mer/7-mer distribution shifted substantially to 7-mer. There is little retention for 3/4-mers and 6-mer in Δ3.2, although there is moderate retention of the 7-mer, likely due to a more stable RNA-DNA hybrid. Right panel: quantitative comparison of RNA retention on bead-immobilized $RP_{ITC<7}$; results reflect mean and SD of four independent experiments.

for the 7-mer), likely due to loss of σ3.2 interactions with parts of the transcription complex that control scrunching and RNA release. These results establish that a substantial portion of the accumulated 6-mer seen on transcription gels is due to RNA stably attached to the transcription complex, as opposed to being released quickly as abortive products.

Promoter Escape Involves Additional RNAP Pausing

All FRET experiments so far were on complexes synthesizing RNAs of up to 7 nt in length. To place our studies in the context of the entire initial transcription up to promoter escape, we performed smFRET on surface-immobilized complexes provided with all four NTPs. Based on our molecular modeling and the DNA conformational changes during escape (Kapanidis et al., 2005b; Mukhopadhyay et al., 2001), we expected a further FRET increase beyond the E*~0.45 state prior to escape due to additional DNA scrunching. This increase was expected to reach a maximum at the point of escape, leading to a FRET decrease when RNAP breaks its promoter contacts and translocates forward by a turn of DNA. After this transition, we expected FRET to stay low (at levels similar to that for RP_o). To avoid any potential interference with the re-annealing of the upstream region of the transcription bubble during escape, we used homoduplex DNA (Figure 6A).

Several time traces showed the pattern expected for escape (Figures 6B and 6C) and exhibited four main features: first,

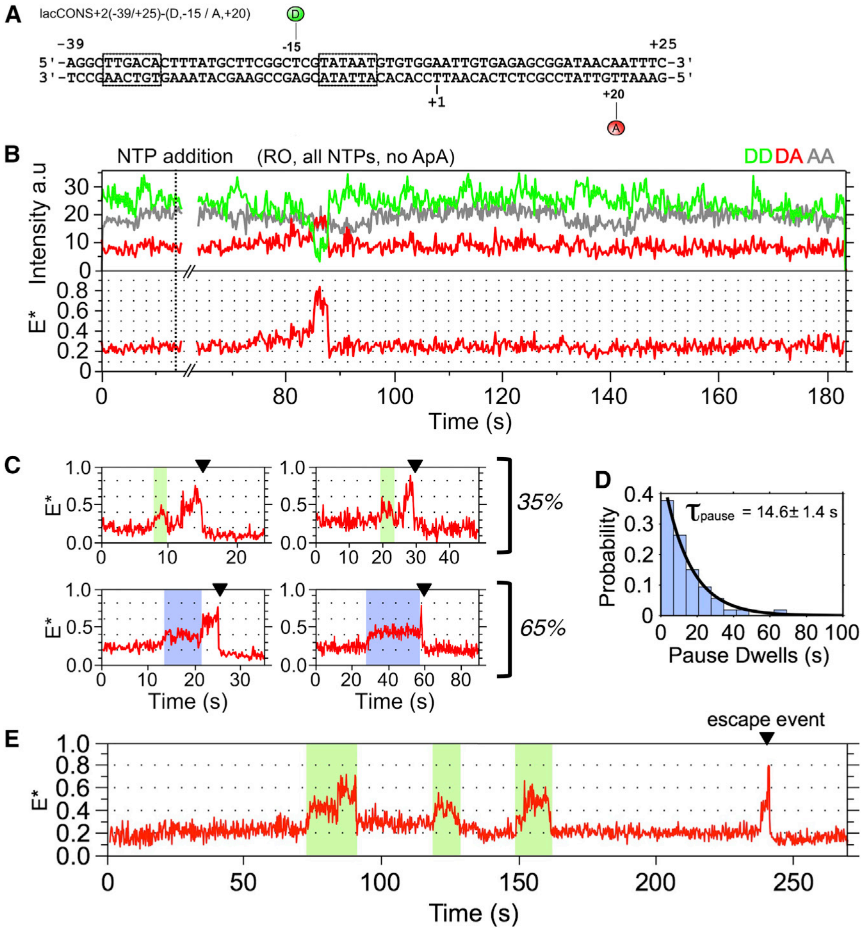

Figure 6. Initiation Pausing on the Path to Promoter Escape

(A) Promoter DNA fragment used.

(B) Time trace showing FRET changes consistent with escape. We added 200 μM ATP and 100 μM UTP, GTP, and CTP. The escape event was marked by the sharp FRET decrease from the maximum FRET state to the baseline (~88 s). No significant E* change is observed after escape during the remaining ~100 s.

(C) Common behaviors consistent with escape. Top panels: example of escapes (marked by black arrowhead) preceded by a clear abortive cycle (green highlight). Bottom panels: examples of escapes preceded by a pause at E*~0.37 (blue highlight).

(D) Dwell-time distribution of pauses in (C) (n = 130).

(E) Time trace showing three abortive cycles (green highlight) followed by a cycle consistent with escape.

See also Figure S4.

upon NTP addition, complexes displayed a FRET increase to a maximum E*of ~0.6–0.8; no such states were seen in $RP_{ITC\leq7}$. Second, in ~50% of such traces, the FRET change included a pause at E* of 0.35–0.4 before reaching E* > 0.6 states (Figure 6C, bottom); the pause lasted for 15 ± 1 s (Figure 6D), similar to that observed in $RP_{ITC\leq7}$, and is clearly not a promoter-proximal paused state (Nickels et al., 2004), since such a state would appear only after formation of a low-FRET state (matching the RP_o baseline), something not observed in our traces. The remainder 50% showed no clear pause, but part of this population almost certainly includes pauses too short to capture given our temporal resolution (200 ms). Third, once the E*~0.6–0.8 FRET state was reached, the complexes remained at that state for ~8 s (Figure S4A) prior to returning to the baseline. Fourth, after returning to the baseline, no subsequent FRET events were observed within our observation span; however, since any observation of cycling is limited by bleaching, we cannot unequivocally define the point of escape.

The long dwell at E*~0.35–0.4 confirmed that the paused state in $RP_{ITC\leq7}$ is a true intermediate on the path to escape. Finally, the long dwell (~8 s) in the maximum FRET state corresponds to a state occupied just before the point where RNAP breaks its promoter interactions during escape; we refer to this pause as the "escape pause."

Most molecules reaching the maximum FRET state (65%) do not go through cycling involving synthesis of >4-nt RNAs (Figure 6C, bottom; we cannot detect all abortive RNAs shorter than 5 nt since they do not stably attach to ITCs). The remainder 35% reached the maximum FRET state after cycling (Figure 6C, top panels; Figure 6E). Notably, most RNAP molecules (~70%) did not escape, despite being provided the full set of NTPs at sufficiently high concentrations (≥100 μM); instead, they appear to be locked in abortive transcription, with ~90% resembling $RP_{ITC\leq7}$ (Figure S4B).

DISCUSSION

A Long Transcriptional Pause on a Promoter Rate-Limited in Initial Transcription

Our results establish that initial transcription on *lac* promoter is not a continuous process, but is interrupted by a long pause ("initiation pause") after RNAP synthesizes a 6-nt RNA. The observation of high levels of a 6-nt RNA (along with the absence of a 5- or 7-nt RNA) in the reaction with all NTPs agrees with early observations on *lac*UV5 (Carpousis and Gralla, 1980; Munson and Reznikoff, 1981). The paused initiation complex on *lac* promoters is thus a true intermediate on the path to elongation.

Due to its long lifetime (~20 s), the initiation pause can be rate-limiting for initial transcription. The pause is substantially longer than open-complex formation at *lac*CONS (~3 s; Revyakin et al., 2004), and comparable to open-complex formation at *lac*UV5 (~10 s at 37°C and ~30 s at 25°C; Buc and McClure, 1985). The initiation pause is comparable to pauses in elongation, such as promoter-proximal pauses (~30 s at 200 μM NTPs on *lac*; Nickels et al., 2004), and the "elemental" pause

(1.5–10 s, depending on GTP concentration; Larson et al., 2014; ~10 s; Hein et al., 2014).

Promoter Dependence of Initiation Pausing

Apart from *lac*, many other promoters are likely to display initiation pausing. For example, Tn5 promoter, also rate-limited in initial transcription, showed accumulation of a 6-nt RNA (Munson and Reznikoff, 1981). Further, removal of σ3.2 caused a marked change in the pattern of short RNAs both on a T7A1cons and a *gal*P1 promoter (Pupov et al., 2014); the longest RNAs eliminated by σ3.2 removal on T7A1cons likely reflect paused ITCs with RNAs equivalent to 5- and 6-nt RNA. The excellent agreement with the length of 6-nt RNA seen on our *lac* promoter supports the presence of initiation pauses in these promoters.

There are, however, promoters linked to limited short RNA transcription prior to escape (e.g., T5N25, *rrn*B); such promoters should exhibit less pausing, whereas promoters limited in initial transcription should exhibit significant initiation pausing. This promoter dependence also implies that although σ3.2 is a major pausing determinant, there are additional, DNA-sequence-dependent determinants that modulate the transition from 6- to 7-nt RNA; this is supported by the fact that σ3.2 removal did not eliminate 6-nt RNA accumulation on *lac*CONS (Figure 5B). It is likely that some of these sequence determinants are present in the initial transcription sequence, since it can drastically change the profile of abortive transcripts (Hsu et al., 2006). Consistent with this, we showed that altering the DNA sequence at positions +6 and +7 to remove a short sequence element ($Y^{-1}G^{+1}$, also a major determinant of elongation pausing; Vvedenskaya et al., 2014; Larson et al., 2014) significantly reduces initiation pausing at *lac* and on many promoters carrying the sequence element (Bauer et al., 2016).

Possible Roles of Initiation Pausing

Initiation pausing can modulate the rate of promoter escape and RNA synthesis. Initiation pausing can also act as a timing delay that increases the spacing between RNAP molecules in elongation, affecting pausing in elongation (Epshtein and Nudler, 2003) and transcription-translation coordination. For some promoters, the combination of multiple rate-limiting steps of similar timescale (e.g., for *lac* promoter, where promoter melting, initiation pause, and promoter-proximal pause last 20–30 s each; Buc and McClure, 1985; Nickels et al., 2004) can turn an exponential distribution of transcription times (i.e., as for a single rate-limiting step) to a distribution with a longer and less variable time delay between RNAPs leaving the promoter. Initiation pausing may also provide more opportunities for regulatory proteins and small molecules to bind ITCs and modulate transcription.

Region σ3.2 Controls Pausing by Transiently Blocking RNA Extension beyond 6 nt

Our work establishes region σ3.2 as a major determinant for initiation pausing and as the structural element that controls the position of initiation pausing. Region σ3.2 interacts with the template strand (positions −3 and −4) and blocks the RNA exit path by clashing with the 5′ end of nascent RNA (Basu et al., 2014; Kulbachinskiy and Mustaev, 2006; Murakami, 2013; Zhang et al., 2012); σ3.2 has also been shown to be a major determinant of abortive initiation (Murakami et al., 2002). Partial removal of σ3.2 changes the distribution of short RNAs (e.g., decreasing the levels of 5- to 9-nt RNAs) at the T7A1cons promoter (Kulbachinskiy and Mustaev, 2006; Pupov et al., 2014). Such changes led to proposals that σ3.2 hinders RNA extension, while its removal allows extension of RNAs that would otherwise abort (e.g., 5- to 9-nt RNAs on T7A1cons).

Our results show σ3.2 indeed acts as the protein element that sets the stage for pausing at RP_{ITC6}; we suggest that the presence of σ3.2 along the path of growing RNA provides an initial time window (linked to σ3.2 repositioning) that allows RNAP to enter paused states, the stability of which is governed by a complex landscape of determinants, including DNA sequence. In short, σ3.2 is the RNAP structural element that *enables* initiation pausing (and consequently, regulation) at the 6-to-7 transition.

Our results also suggest that σ3.2 stabilizes the scrunched conformation in RP_{ITC6}, with stabilization seen first when RNA reaches 5 nt in length. One possibility for the stabilization is that the 5′ end of RNA interacts with σ3.2, as suggested by ITC structures (Basu et al., 2014; Zuo and Steitz, 2015); since the structures showed σ3.2 in slightly different conformations, these conformations may be linked with different pause-recovery kinetics. Interactions between template and σ3.2 may also prevent lateral movements of the template strand that would otherwise allow RNA to backtrack and be released more easily (see Discussion on backtracking; Pupov et al., 2014); consistent with this, a Δ3.2 mutant exhibits faster bubble dynamics in RP_o (D.D. and A.N.K., unpublished data).

Backtracking and Abortive Release Mechanism

Our FRET results on RP_{ITC7} revealed transitions consistent with scrunching relaxation by RNA backtracking, since the relaxed state matches the FRET signature of the paused state in RP_{ITC6}, which is likely to be in its pre-translocated state. In the backtracked RP_{ITC7} state, RNAP is inactive, since its active site is blocked by the 3′ end of RNA; this state also leads to RNA loss. These series of transitions suggest that the backtracked state is an intermediate on the path to RNA release. RNA backtracking in initiation is supported by reports showing that transcript-cleavage factor GreA (which cleaves the 3′ end of RNA in backtracked elongation complexes to generate new extensible 3′ ends) alters the abortive products on T7A1 and T5N25anti (Feng et al., 1994; Hsu et al., 1995), as well as by in vivo work suggesting that the main GreA role is to relieve transcriptional arrest at specific promoters before promoter clearance (Stepanova et al., 2007). These findings support a model wherein short RNAs are displaced from the active center in a backward direction, form backtracked states (wherein the 3′ end of RNA frays from the template and enters the secondary channel), and get released (Feng et al., 1994; Hsu, 2009; Hsu et al., 1995; Stepanova et al., 2007).

Initial Transcription and Promoter Escape

We also observed DNA conformational changes occurring between the 6-mer pause and promoter escape. Notably, we observed a pause just before escape ("escape pause"), where the maximum scrunching is expected to be reached; this pause may reflect destabilization of contacts between σ region 4 (σ4)

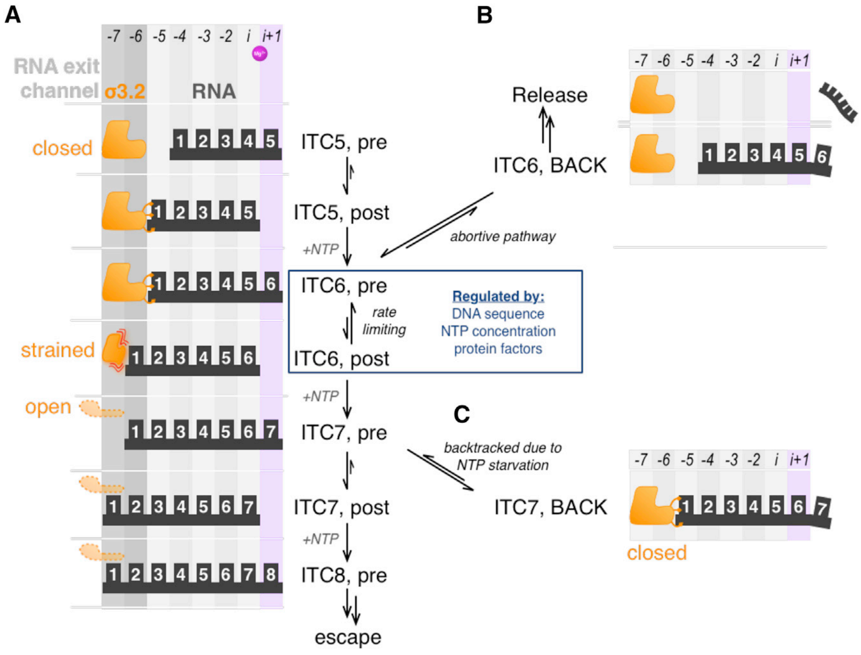

Figure 7. A Working Model for Initial Transcription

(A) Productive path for initial transcription. Colored columns show translocational registers adopted by growing RNA (in black). Binding site for incoming NTP is in light purple; σ3.2 loop is shown in three putative conformations (in orange). The translocational equilibrium for RP_{ITC6} is controlled by several regulatory factors that modulate the lifetime of paused states arising from a pre-translocated RP_{ITC6}.

(B) Abortive path for initial transcription, branching from the pre-translocated RP_{ITC6} state of the productive path.

(C) Path for the formation of stable backtracked scrunched states, branching from the pre-translocated RP_{ITC6} state of initial transcription during NTP starvation that limits RNA synthesis to 7 nt in length.

and the −35 element (Vassylyev et al., 2002) or the last stage of σ3.2 displacement from the RNA exit channel, an event that affects σ4-promoter interactions (Mekler et al., 2002; Murakami and Darst, 2003; Murakami et al., 2002; Vassylyev et al., 2002). The escape pause presents another rate-limiting step with regulation potential.

Heterogeneity of ITCs

Our results showed that active complexes exhibit heterogeneity, since ITCs imaged under identical conditions displayed varying tendencies for abortive cycling (Figure 4A). The heterogeneity was long lived, with "stably scrunched" or "cycling" behaviors persisting for >10 min. Such functional heterogeneity has been seen in elongation (Herbert et al., 2006) and may reflect the presence of moribund abortive complexes (Hsu, 2002; Kubori and Shimamoto, 1996) that could underpin a mode of regulation; e.g., regulatory molecules or different promoters may affect the distribution between behaviors, altering the probability of producing full-length RNA. The heterogeneity source is unclear, but it may reflect static conformational heterogeneity between molecules, as well as compositional differences between molecules, due to translation errors or chemical changes occurring either in vivo or during RNAP preparation, as suggested for elongation (Larson et al., 2011).

A Working Model for Initial Transcription

Based on our findings and existing literature, we present a working model for initial transcription that includes initiation pausing as a regulatory checkpoint controlled by structural, sequence, and environmental factors (Figure 7). While the model focuses on *lac*, many features should apply to most bacterial promoters.

Initial transcription starts with synthesis of RNAs 2–4 nt in length, accompanied by increasing scrunching; these products dissociate quickly (Carpousis and Gralla, 1980). When the RNA reaches 5 nt in length, it is stabilized in RP_{ITC5}, most likely at its post-translocated state. This frees the i+1 site at the active center for binding the next complementary NTP, which is incorporated quickly to form a pre-translocated RP_{ITC6} (as seen in our results and in a complex resembling RP_{ITC6}; Basu et al., 2014). The presence of σ3.2-template interactions limits initial scrunching to 4 nt (i.e., up to the initiation pause) in the template and non-template strands. The 5′ end of the 6-nt RNA clashes with σ3.2, hindering template/RNA translocation from the pre- to post-translocated state.

At this point, and in a way akin to "ubiquitous" pausing in elongation (Herbert et al., 2006), the complex enters an off-pathway paused state. The lifetime of pausing is modulated by several determinants (such as DNA sequence, nucleotide identity and concentration, and protein factors); this multi-partite modulation effectively controls the kinetics of the transition from the pre- to post-translocational register of RP_{ITC6} and regulates initial transcription.

At *lac*, the overall context biases the translocational balance toward a pre-translocated RP_{ITC6} and a paused state lasting for 15–25 s. In productive initial transcription, GTP binds to a transiently sampled post-translocated state of RP_{ITC6} and extends RNA to a 7-mer. Although our results point to the translocation step being rate-limiting, we cannot exclude that NTP binding and incorporation may also be affected, as in pauses without backtracking (Kireeva and Kashlev, 2009). The formation of a 7-mer stabilizes RP_{ITC7} and allows translocation to the post-translocated state, where the RNA exit channel entrance is kept open by the 5′ end of RNA. Ultimately, σ3.2 is displaced by the growing RNA, weakening σ^{70}-promoter contacts and driving promoter escape. The growing RNA also severs the contacts of σ3.2 with the template, allowing the template to scrunch further up to promoter escape. The evidence for backtracking in the case of NTP starvation (due to the use of NTP subsets) also identifies the secondary channel as the likely RNA release route.

Relevance to Other Transcription Systems

Since the negative charge of σ3.2 is highly conserved in alternative σ factors, initiation pausing may be present in non-σ^{70} bacterial promoters (Pupov et al., 2014). The conservation of the σ3.2 loop structural feature in eukaryotes and archaea raises the possibility of initiation pausing in a diverse range of organisms, e.g., due to the TFIIB B-finger (Sainsbury et al., 2013) or a similar structure in archaeal TFB; the latter has already been shown to increase abortive transcription when added to a transcribing archaeal RNAP (Werner and Weinzierl, 2005).

EXPERIMENTAL PROCEDURES

DNA, RNAP, and RP_o Preparation

Labeled oligos were purchased from IBA. WT RNAP core from *E. coli* with a His-tag at the β′ C terminus was prepared as described (Belogurov et al., 2007). WT and mutant σ^{70} lacking residues 513–519 (Δ3.2) were purified as described (Kulbachinskiy and Mustaev, 2006). WT and Δ3.2 holoenzymes were prepared by incubating 50 nM RNAP core with 250 nM σ^{70} for 30 min at 33°C. RP_o was formed by incubating RNAP holoenzyme with DNA followed by heparin challenge (Kapanidis et al., 2006). For rifampicin experiments, 250 nM rifampicin was incubated with RNAP for 30 min at 33°C before DNA was added.

In Vitro Transcription

Reactions were performed as described (Cordes et al., 2010; Robb et al., 2013) with modifications to mimic our smFRET experiments. Reactions were initiated by mixing 1 μL RP_o with a 4 μL mix containing 4 U RNAsin, 0.1 mg/mL heparin, and the relevant NTP mixture in 1× KG7 buffer (40 mM HEPES-NaOH [pH 7], 100 mM potassium glutamate, 10 mM $MgCl_2$, 100 μg/mL BSA, 1 mM DTT, and 5% glycerol). NTPs and ApA were added at 80 and 500 μM, respectively. Reactions were supplemented with [α^{32}P]UTP (0.6 μCi/μL, PerkinElmer), incubated for 10–60 s at 21°C, stopped by 7.5 μL of 1 M HCl, and neutralized with Tris/EDTA (Malinen et al., 2015). The reactions were precipitated and kept at −20°C. Pellets were dried, dissolved in loading dye, and incubated for 4 min at 95°C before gel electrophoresis and autoradiography.

For transcription on beads, RNAP was assembled in 10 μL transcription buffer (TB) (40 mM HEPES [pH 8.0], 50 mM NaCl, 5 mM $MgCl_2$, and 5% glycerol) and incubated with 10 μL Ni^{2+} agarose beads; samples were centrifuged and 6 μL TB was discarded. One microliter *lac*UV5 DNA was added and incubated for 10 min at 37°C. Transcription was initiated by 1 μL of 5 mM ApA; 2 μL of 250 μM GTP, UTP (to 31 μM final), and 0.6 μCi [^{32}P]-UTP per reaction; and incubation for 20 s at 37°C. Reactions were stopped by washing the complexes; the supernatant was discarded and Ni beads were supplemented with stop solution. Samples were incubated for 2 min at 65°C before being loaded on a PAGE denaturing gel.

Single-Molecule FRET

TIRF experiments with alternating-laser excitation (Kapanidis et al., 2004) were performed on a custom microscope (Holden et al., 2010). To immobilize RP_o, 10 nM biotinylated penta-His antibody was incubated for 10 min on a neutravidin-coated surface; unbound antibodies were removed, and 1 nM RP_o was added and incubated for 5 min. Once RP_o was immobilized, KG7 imaging buffer (40 mM HEPES-NaOH [pH 7], 100 mM potassium glutamate, 10 mM $MgCl_2$, 1 mm DTT, 100 μg/mL BSA, 5% glycerol, and 2 mM Trolox) and an oxygen scavenging system (1 mg/mL glucose oxidase, 40 μg/mL catalase, and 1.4% w/v D-glucose) were added.

To form RP_{ITC} synthesizing RNAs up to N nt in length ($RP_{ITC\leq N}$), NTP reaction mixtures were added manually during acquisition; unless stated otherwise, the final NTP concentration was 80 μM. For $RP_{ITC\leq 4}$, the NTP mixture consisted of imaging buffer plus UTP. For $RP_{ITC\leq 5}$, 3′ dGTP (TriLink BioTechnologies) was added to $RP_{ITC\leq 4}$ mixture. For $RP_{ITC\leq 7}$, GTP was added to $RP_{ITC\leq 4}$ mixture. For promoter escape, the imaging buffer was supplemented with ATP at 200 μM, and UTP, GTP, and CTP at 100 μM.

Fluorescence intensities were extracted using *twoTone* (Holden et al., 2010), and the uncorrected FRET efficiency (E*) was calculated as described (Pinkney et al., 2012). To select traces, we used well-defined criteria (see Supplemental Information). The dwell times of scrunched states were extracted via hidden Markov modeling (HMM) analysis (Le Reste et al., 2012) and fitted with exponentials to extract dwell times.

For extended protocols, see Supplemental Experimental Procedures.

SUPPLEMENTAL INFORMATION

Supplemental Information includes Supplemental Experimental Procedures and four figures and can be found with this article online at http://dx.doi.org/10.1016/j.molcel.2016.08.011.

AUTHOR CONTRIBUTIONS

A.N.K. conceived and supervised the project. A.N.K., D.D., D.L.V.B., and K.B. designed experiments. D.D., L.F., N.R., and P.Z. performed microscopy measurements. L.C.H. and K.G. performed preliminary microscopy experiments. D.D., D.L.V.B., G.E., K.G., N.R., P.Z., and A.N.K. performed data analysis. D.L.V.B., N.R., A.T., and Z.M. performed biochemical assays. D.D., D.L.V.B., and A.N.K. wrote the manuscript.

ACKNOWLEDGMENTS

We thank Dr. Javier Periz for help with protein purification and Drs. David Dulin and Anssi Malinen for insightful discussions. D.D. was supported by a UK EPSRC DTA studentship. D.L.V.B. was supported by an NSF postdoctoral fellowship (DBI-1309306) and an EPAC Junior Research Fellowship (Linacre College, Oxford). K.G. was supported by a Clarendon scholarship (Oxford University). G.E. was supported by the Life Sciences Interface DTC. Work in the A.N.K. lab was supported by the ERC (grant 261227), the UK BBSRC (grants BB/H01795X/1 and BB/J00054X/1), and the Wellcome Trust (grant 110164/Z/15/Z).

Received: May 22, 2015
Revised: April 12, 2016
Accepted: August 5, 2016
Published: September 8, 2016

REFERENCES

Basu, R.S., Warner, B.A., Molodtsov, V., Pupov, D., Esyunina, D., Fernández-Tornero, C., Kulbachinskiy, A., and Murakami, K.S. (2014). Structural basis of transcription initiation by bacterial RNA polymerase holoenzyme. J. Biol. Chem. *289*, 24549–24559.

Bauer, D.L.V., Duchi, D., and Kapanidis, A.N. (2016). *E.coli* RNA polymerase pauses during initial transcription. Biophys. J. *110* (*Supplement 1*), 21a, http://dx.doi.org/10.1016/j.bpj.2015.11.170.

Belogurov, G.A., Vassylyeva, M.N., Svetlov, V., Klyuyev, S., Grishin, N.V., Vassylyev, D.G., and Artsimovitch, I. (2007). Structural basis for converting a general transcription factor into an operon-specific virulence regulator. Mol. Cell *26*, 117–129.

Brodolin, K., Zenkin, N., Mustaev, A., Mamaeva, D., and Heumann, H. (2004). The sigma 70 subunit of RNA polymerase induces lacUV5 promoter-proximal pausing of transcription. Nat. Struct. Mol. Biol. *11*, 551–557.

Buc, H., and McClure, W.R. (1985). Kinetics of open complex formation between Escherichia coli RNA polymerase and the lac UV5 promoter. Evidence for a sequential mechanism involving three steps. Biochemistry *24*, 2712–2723.

Campbell, E.A., Korzheva, N., Mustaev, A., Murakami, K., Nair, S., Goldfarb, A., and Darst, S.A. (2001). Structural mechanism for rifampicin inhibition of bacterial RNA polymerase. Cell *104*, 901–912.

Carpousis, A.J., and Gralla, J.D. (1980). Cycling of ribonucleic acid polymerase to produce oligonucleotides during initiation in vitro at the lac UV5 promoter. Biochemistry *19*, 3245–3253.

Cordes, T., Santoso, Y., Tomescu, A.I., Gryte, K., Hwang, L.C., Camará, B., Wigneshweraraj, S., and Kapanidis, A.N. (2010). Sensing DNA opening in transcription using quenchable Förster resonance energy transfer. Biochemistry *49*, 9171–9180.

Epshtein, V., and Nudler, E. (2003). Cooperation between RNA polymerase molecules in transcription elongation. Science *300*, 801–805.

Feng, G.H., Lee, D.N., Wang, D., Chan, C.L., and Landick, R. (1994). GreA-induced transcript cleavage in transcription complexes containing Escherichia coli RNA polymerase is controlled by multiple factors, including nascent transcript location and structure. J. Biol. Chem. *269*, 22282–22294.

Gralla, J.D., Carpousis, A.J., and Stefano, J.E. (1980). Productive and abortive initiation of transcription in vitro at the lac UV5 promoter. Biochemistry *19*, 5864–5869.

Hein, P.P., Kolb, K.E., Windgassen, T., Bellecourt, M.J., Darst, S.A., Mooney, R.A., and Landick, R. (2014). RNA polymerase pausing and nascent-RNA structure formation are linked through clamp-domain movement. Nat. Struct. Mol. Biol. *21*, 794–802.

Herbert, K.M., La Porta, A., Wong, B.J., Mooney, R.A., Neuman, K.C., Landick, R., and Block, S.M. (2006). Sequence-resolved detection of pausing by single RNA polymerase molecules. Cell *125*, 1083–1094.

Holden, S.J., Uphoff, S., Hohlbein, J., Yadin, D., Le Reste, L., Britton, O.J., and Kapanidis, A.N. (2010). Defining the limits of single-molecule FRET resolution in TIRF microscopy. Biophys. J. *99*, 3102–3111.

Hsu, L.M. (2002). Promoter clearance and escape in prokaryotes. Biochim. Biophys. Acta *1577*, 191–207.

Hsu, L.M. (2009). Monitoring abortive initiation. Methods *47*, 25–36.

Hsu, L.M., Vo, N.V., and Chamberlin, M.J. (1995). Escherichia coli transcript cleavage factors GreA and GreB stimulate promoter escape and gene expression in vivo and in vitro. Proc. Natl. Acad. Sci. USA *92*, 11588–11592.

Hsu, L.M., Cobb, I.M., Ozmore, J.R., Khoo, M., Nahm, G., Xia, L., Bao, Y., and Ahn, C. (2006). Initial transcribed sequence mutations specifically affect promoter escape properties. Biochemistry *45*, 8841–8854.

Kapanidis, A.N., Lee, N.K., Laurence, T.A., Doose, S., Margeat, E., and Weiss, S. (2004). Fluorescence-aided molecule sorting: analysis of structure and interactions by alternating-laser excitation of single molecules. Proc. Natl. Acad. Sci. USA *101*, 8936–8941.

Kapanidis, A.N., Laurence, T.A., Lee, N.K., Margeat, E., Kong, X., and Weiss, S. (2005a). Alternating-laser excitation of single molecules. Acc. Chem. Res. *38*, 523–533.

Kapanidis, A.N., Margeat, E., Laurence, T.A., Doose, S., Ho, S.O., Mukhopadhyay, J., Kortkhonjia, E., Mekler, V., Ebright, R.H., and Weiss, S. (2005b). Retention of transcription initiation factor sigma70 in transcription elongation: single-molecule analysis. Mol. Cell *20*, 347–356.

Kapanidis, A.N., Margeat, E., Ho, S.O., Kortkhonjia, E., Weiss, S., and Ebright, R.H. (2006). Initial transcription by RNA polymerase proceeds through a DNA-scrunching mechanism. Science *314*, 1144–1147.

Kireeva, M.L., and Kashlev, M. (2009). Mechanism of sequence-specific pausing of bacterial RNA polymerase. Proc. Natl. Acad. Sci. USA *106*, 8900–8905.

Kubori, T., and Shimamoto, N. (1996). A branched pathway in the early stage of transcription by Escherichia coli RNA polymerase. J. Mol. Biol. *256*, 449–457.

Kulbachinskiy, A., and Mustaev, A. (2006). Region 3.2 of the sigma subunit contributes to the binding of the 3′-initiating nucleotide in the RNA polymerase active center and facilitates promoter clearance during initiation. J. Biol. Chem. *281*, 18273–18276.

Larson, M.H., Landick, R., and Block, S.M. (2011). Single-molecule studies of RNA polymerase: one singular sensation, every little step it takes. Mol. Cell *41*, 249–262.

Larson, M.H., Mooney, R.A., Peters, J.M., Windgassen, T., Nayak, D., Gross, C.A., Block, S.M., Greenleaf, W.J., Landick, R., and Weissman, J.S. (2014). A pause sequence enriched at translation start sites drives transcription dynamics in vivo. Science *344*, 1042–1047.

Le Reste, L., Hohlbein, J., Gryte, K., and Kapanidis, A.N. (2012). Characterization of dark quencher chromophores as nonfluorescent acceptors for single-molecule FRET. Biophys. J. *102*, 2658–2668.

Malinen, A.M., Turtola, M., and Belogurov, G.A. (2015). Monitoring translocation of multisubunit RNA polymerase along the DNA with fluorescent base analogues. Methods Mol. Biol. *1276*, 31–51.

Margeat, E., Kapanidis, A.N., Tinnefeld, P., Wang, Y., Mukhopadhyay, J., Ebright, R.H., and Weiss, S. (2006). Direct observation of abortive initiation and promoter escape within single immobilized transcription complexes. Biophys. J. *90*, 1419–1431.

McClure, W.R., and Cech, C.L. (1978). On the mechanism of rifampicin inhibition of RNA synthesis. J. Biol. Chem. *253*, 8949–8956.

Mekler, V., Kortkhonjia, E., Mukhopadhyay, J., Knight, J., Revyakin, A., Kapanidis, A.N., Niu, W., Ebright, Y.W., Levy, R., and Ebright, R.H. (2002). Structural organization of bacterial RNA polymerase holoenzyme and the RNA polymerase-promoter open complex. Cell *108*, 599–614.

Mukhopadhyay, J., Kapanidis, A.N., Mekler, V., Kortkhonjia, E., Ebright, Y.W., and Ebright, R.H. (2001). Translocation of σ(70) with RNA polymerase during transcription: fluorescence resonance energy transfer assay for movement relative to DNA. Cell *106*, 453–463.

Munson, L.M., and Reznikoff, W.S. (1981). Abortive initiation and long ribonucleic acid synthesis. Biochemistry *20*, 2081–2085.

Murakami, K.S. (2013). X-ray crystal structure of Escherichia coli RNA polymerase σ70 holoenzyme. J. Biol. Chem. *288*, 9126–9134.

Murakami, K.S., and Darst, S.A. (2003). Bacterial RNA polymerases: the wholo story. Curr. Opin. Struct. Biol. *13*, 31–39.

Murakami, K.S., Masuda, S., and Darst, S.A. (2002). Structural basis of transcription initiation: RNA polymerase holoenzyme at 4 Å resolution. Science *296*, 1280–1284.

Nickels, B.E., Mukhopadhyay, J., Garrity, S.J., Ebright, R.H., and Hochschild, A. (2004). The sigma 70 subunit of RNA polymerase mediates a promoter-proximal pause at the lac promoter. Nat. Struct. Mol. Biol. *11*, 544–550.

Pinkney, J.N.M., Zawadzki, P., Mazuryk, J., Arciszewska, L.K., Sherratt, D.J., and Kapanidis, A.N. (2012). Capturing reaction paths and intermediates in Cre-loxP recombination using single-molecule fluorescence. Proc. Natl. Acad. Sci. USA *109*, 20871–20876.

Pupov, D., Kuzin, I., Bass, I., and Kulbachinskiy, A. (2014). Distinct functions of the RNA polymerase σ subunit region 3.2 in RNA priming and promoter escape. Nucleic Acids Res. *42*, 4494–4504.

Revyakin, A., Ebright, R.H., and Strick, T.R. (2004). Promoter unwinding and promoter clearance by RNA polymerase: detection by single-molecule DNA nanomanipulation. Proc. Natl. Acad. Sci. USA *101*, 4776–4780.

Revyakin, A., Liu, C., Ebright, R.H., and Strick, T.R. (2006). Abortive initiation and productive initiation by RNA polymerase involve DNA scrunching. Science *314*, 1139–1143.

Robb, N.C., Cordes, T., Hwang, L.C., Gryte, K., Duchi, D., Craggs, T.D., Santoso, Y., Weiss, S., Ebright, R.H., and Kapanidis, A.N. (2013). The transcription bubble of the RNA polymerase-promoter open complex exhibits conformational heterogeneity and millisecond-scale dynamics: implications for transcription start-site selection. J. Mol. Biol. *425*, 875–885.

Saecker, R.M., Record, M.T., Jr., and Dehaseth, P.L. (2011). Mechanism of bacterial transcription initiation: RNA polymerase-promoter binding, isomerization to initiation-competent open complexes, and initiation of RNA synthesis. J. Mol. Biol. *412*, 754–771.

Sainsbury, S., Niesser, J., and Cramer, P. (2013). Structure and function of the initially transcribing RNA polymerase II-TFIIB complex. Nature *493*, 437–440.

Stepanova, E., Lee, J., Ozerova, M., Semenova, E., Datsenko, K., Wanner, B.L., Severinov, K., and Borukhov, S. (2007). Analysis of promoter targets

for Escherichia coli transcription elongation factor GreA in vivo and in vitro. J. Bacteriol. *189*, 8772–8785.

Vassylyev, D.G., Sekine, S., Laptenko, O., Lee, J., Vassylyeva, M.N., Borukhov, S., and Yokoyama, S. (2002). Crystal structure of a bacterial RNA polymerase holoenzyme at 2.6 Å resolution. Nature *417*, 712–719.

Vvedenskaya, I.O., Vahedian-Movahed, H., Bird, J.G., Knoblauch, J.G., Goldman, S.R., Zhang, Y., Ebright, R.H., and Nickels, B.E. (2014). Interactions between RNA polymerase and the "core recognition element" counteract pausing. Science *344*, 1285–1289.

Werner, F., and Weinzierl, R.O.J. (2005). Direct modulation of RNA polymerase core functions by basal transcription factors. Mol. Cell. Biol. *25*, 8344–8355.

Zhang, Y., Feng, Y., Chatterjee, S., Tuske, S., Ho, M.X., Arnold, E., and Ebright, R.H. (2012). Structural basis of transcription initiation. Science *338*, 1076–1080.

Zuo, Y., and Steitz, T.A. (2015). Crystal structures of the E. coli transcription initiation complexes with a complete bubble. Mol. Cell *58*, 534–540.

Molecular Cell, Volume *63*

Supplemental Information

RNA Polymerase Pausing during Initial Transcription

Diego Duchi, David L.V. Bauer, Laurent Fernandez, Geraint Evans, Nicole Robb, Ling Chin Hwang, Kristofer Gryte, Alexandra Tomescu, Pawel Zawadzki, Zakia Morichaud, Konstantin Brodolin, and Achillefs N. Kapanidis

SUPPLEMENTAL EXPERIMENTAL PROCEDURES

DNA and protein preparation. Oligonucleotides labelled with Cy3B and ATTO647N were purchased from IBA Life Sciences (Germany). WT RNAP core from *E. coli* with a His-tag at the C-terminal end of the β' subunit was prepared as described (Belogurov et al., 2007). WT σ^{70} and mutant σ^{70} lacking residues 513-519 (Δ3.2) were both purified as described (Kulbachinskiy and Mustaev, 2006; Tupin et al., 2010). The N-terminal 6xHis tag was removed by thrombin cleavage kit. WT and Δ3.2 holoenzymes were prepared by incubating 50 nM RNAP core with 250 nM σ^{70} for 30 min at 33°C.

RP_O formation. RP_O was formed as previously described (Kapanidis et al., 2006; Margeat et al., 2006). Briefly, 10 nM of the promoter DNA fragment was incubated with 50 nM *E. coli* RNAP holoenzyme in KG7 buffer consisting of 40 mM HEPES-NaOH (pH 7), 100 mM potassium glutamate, 10 mM $MgCl_2$, 100 µg/mL BSA, 1 mM DTT, and 5% glycerol to give a final volume of 20 µL. The mixture was incubated at 37°C for 15 min, after which 1 mg/mL heparin sepharose (GE Healthcare) was added to disrupt non-specific RNAP-promoter DNA complexes. The mixture was incubated at 37°C for 30 s and then centrifuged to remove sepharose beads; subsequently, 13 µL of the supernatant was removed and transferred to a pre-warmed tube and incubated for a further 20 min at 37°C. For rifampicin experiments, 250 nM rifampicin was incubated with RNAP for 30 min at 33°C before DNA was added. Then, we followed the RP_O formation protocol described above.

***In vitro* transcription (IVT) reactions**. *In vitro* transcription reactions were performed as described (Cordes et al., 2010; Robb et al., 2013), with minor modifications designed to mimic our smFRET experimental conditions. RP_O was formed with 50 nM promoter DNA and 250 nM holoenzyme in 1x KG7 buffer. *In vitro* transcription reactions were initiated by mixing 1 µL of the RP_O mixture with 4 µL reaction mix containing 4 units of RNAsin (Promega, USA), 0.1 mg/mL heparin, and the relevant NTP mixture in 1x KG7 buffer. Details of the NTP mixtures are given in the relevant figure captions. In all cases however, NTPs were added to a final concentration of 80 µM, and ApA was added to a final concentration of 500 µM. Reaction mixes were supplemented with [α^{32}P]UTP (10 µCi/µL, PerkinElmer) and was added at 0.6 µCi/µL. Reactions were incubated for 10-60 s at 21°C, and stopped by the addition of 7.5 µL of 1M HCl, which was then neutralized with Tris/EDTA (Malinen et al., 2015). The reactions were precipitated using glycogen as a carrier (Hsu, 2009) and kept overnight at -20 ºC. Pellets were dried and then taken up in 10 µL of loading dye (90% formamide, 10 mM EDTA, amaranth dye). Mixtures were incubated for 4 min at 95°C before being loaded on a 7 M urea, 20% polyacrylamide sequencing gel and visualized by autoradiography.

***In vitro* transcription on bead-immobilized transcription complexes.** The RNAP holoenzyme was assembled by mixing 200 nM core RNAP and 1 µM σ subunit in 10 µl TB (40 mM HEPES pH 8.0, 50 mM NaCl, 5 mM $MgCl_2$; 5% glycerol) and incubating for 5 min at 37°C. The mixtures were incubated with 10µL Ni^{2+} agarose beads for 5 min at 25°C with shaking at 800 rpm. Samples were centrifuged and 6 µL of TB was discarded. One µl of a 116-bp *lac*UV5 promoter fragment (promoter positions -59 to +58, see Morichaud et al., 2016) was added to 40 nM final and incubated for 10 min at 37°C. Transcription was initiated by adding 1 µl 5 mM ApA, followed by 2 µl of 250 µM GTP, UTP (to 31 µM final) and 0,6 µCi [^{32}P]-UTP per reaction, and incubation for 20 s at 37°C. Reactions were stopped by washing the complexes with 0.5 ml of TB for 30 s; after centrifugation (30 s), the supernatant was discarded and Ni-agarose beads were supplemented with stop solution; the time from the end of the transcription reaction to the addition of stop solution was ~2 min. The sample volumes in the "input" and "wash" reactions were adjusted to be equal. Samples were then incubated for 2 min at 65°C before being loaded on a 24% PAGE-7M urea denaturing gel. Gels were scanned with Typhoon 9400 Imager (GE Healthcare) and quantified using ImageQuant software.

Instrumentation. Single-molecule TIRF experiments were performed on a custom built objective-type TIRF microscope. A green (532 nm; Cobolt Samba) and a red (635 nm; Cube Coherent) laser were combined using a dichroic mirror and then coupled into a fiber optic cable. The fiber output was focussed into the back focal plane of the objective (100× oil-immersion, numerical aperture 1.4, Olympus) and displaced off the optical axis such that the laser light was incident at the slide-solution interface at an angle greater than the critical angle, creating an evanescent wave. ALEX (Kapanidis et al., 2004) was implemented by directly modulating the two lasers. Data was acquired at different alternation rates depending on the experiment. Alternation rates are given in figure legends. Fluorescence emission was collected from the objective and separated from the excitation light by a dichroic mirror (545 nm/650 nm, Semrock) and clean-up filters (545 nm LP, Chroma; and 633/25 nm notch filter, Semrock). The emission signal was focussed on a slit to crop the image and then spectrally separated (using a 630 nm DRLP dichroic; Omega) into donor and acceptor emission channels, which were focused side-by-side onto an EMCCD camera (Andor iXon 897). A CRIFF (ASI) autofocus system was used to ensure focus stability during experiments**.**

Single-molecule FRET experiments. A 10 nM solution of biotinylated penta-His antibody (Qiagen) was incubated for 10 min on a surface coated with NeutrAvidin (Thermo Scientific) to allow binding. After the incubation, excess unbound antibodies were washed away, and a 1 nM solution of pre-formed RP_O was added and incubated for 5 min. Binding was monitored until ~60 molecules were deposited on the surface, and excess unbound complexes were washed away. Once RP_O was immobilized onto the antibody-coated surface, KG7 imaging buffer consisting of 40 mM HEPES-NaOH (pH 7), 100 mM potassium glutamate, 10 mM $MgCl_2$, 1 mm DTT, 100 µg/mL BSA, 5% glycerol, 2 mM UV-treated Trolox, and an oxygen scavenging system (1 mg/mL glucose oxidase, 40 µg/mL catalase, 1.4% w/v D-

glucose) was added. For abortive initiation, the imaging buffer was supplemented with 500 μM ApA dinucleotide (Kapanidis et al., 2006; Margeat et al., 2006). Where indicated, the imaging buffer was supplemented with 500 nM rifampicin.

To form RP_{ITC} engaged in abortive synthesis of RNA products up to N-nt in length ($RP_{ITC\leq N}$), an NTP reaction mixture was added to the observation chamber manually during data acquisition. The NTP reaction mixture varied for different experiments; however, unless stated otherwise, the final concentration of NTPs in the observation chamber was 80 μM. For $RP_{ITC\leq 4}$ experiments the NTP reaction mixture consisted of the imaging buffer supplemented with UTP. For $RP_{ITC\leq 5}$ experiments, 3'-dGTP (TriLink BioTechnologies) was added to the $RP_{ITC\leq 4}$ mixture. For $RP_{ITC\leq 7}$ experiments, GTP was added to the $RP_{ITC\leq 4}$ mixture.

For promoter-escape experiments, the NTP reaction mixture consisted of imaging buffer supplemented with all four NTPs. The final concentration of ATP in the observation chamber was 200 μM, while that of UTP, GTP and CTP was 100 μM. The CRIFF autofocusing system (ASI) was used during data acquisition to ensure focal stability.

The frame rates used for experiments are given in the relevant figure legends. For 20-ms frame rate experiments, excitation powers of 8 mW (532 nm laser) and 4 mW (635 nm laser) were used. For 200-ms frame rate experiments, excitation powers of 0.5 mW (532 nm laser) and 0.15 mW (635 nm laser) were used. All experiments were performed at 21°C.

Data analysis and visualisation. Fluorescence intensities were extracted from EMCCD images using *twoTone* software (Holden et al., 2010). The uncorrected FRET efficiency (E*) was then calculated as described (Pinkney et al., 2012). We manually inspected intensity time trajectories and selected molecules for analysis according to the following criteria: 1) bleached in a single step; 2) circular PSF across DD, DA, and AA channels; 3) no donor photoblinking (although fluctuations were permitted); 4) no acceptor photoblinking, although we allowed for different ATTO647N states (Ha and Tinnefeld, 2012); 5) fluorescence intensities being within a limited range; 6) no defocusing; and 7) no nearby molecules as measured using a nearest-neighbour criterion.

We manually inspected the intensity time trajectories of molecules in abortive initiation experiments and classified them into groups. The baseline state was assigned to RP_O and any increase in FRET that surpassed E*=0.3 was assigned to an initial transcribing complex with the transcription bubble in a scrunched conformation. We refer to these FRET states as scrunched FRET states. Time trajectories showing a stable scrunched state signal that lasted for >120 s were classified as stably scrunched. Time trajectories showing cyclical increases and decreases in FRET between the baseline and the scrunched FRET states were classified as cycling events in which RNA is synthesized during the increase in FRET, and released as abortive RNA during the decrease in FRET. Molecules showing a stepped increase in FRET, where the step lasted for >4 frames were classified as pausing molecules. Molecules showing a decrease in FRET from a maximally scrunched FRET state (excursion state) to a scrunched state above the baseline that lasted for >4 frames were classified as backtracked molecules. Molecules that bleached within 120 s of the addition of the NTP reaction mixture were excluded from analysis, since they could not be classified as either stably scrunched or cycling. The maximum FRET value reached in an $RP_{ITC\leq N}$ experiment was assigned as the FRET value corresponding to RNA molecules N-nt in length (see *Results*). For promoter-escape experiments, we identified specific patterns of signal changes that we assigned to promoter-escape events (see *Results*). For both abortive initiation and promoter escape experiments, molecules exhibiting confounding photophysical fluctuations were excluded from analysis.

HMM Analysis of cycling smFRET time trajectories. HMM analysis was performed on time trajectories displaying dynamic FRET fluctuations using custom-written MATLAB software (Le Reste et al., 2012; Uphoff et al., 2011). For abortive-initiation experiments, each time trajectory was fitted with 1 to 3 states and the best model (number of states) was selected automatically using maximum evidence criteria (Bronson et al., 2009; Uphoff et al., 2011). The FRET efficiency states above the baseline were classified as scrunched states. The dwell times of the scrunched states were extracted and used to generate dwell time distributions, which were fitted with an exponential decay curve. The mean scrunched-state dwell times were extracted from the fits.

HMM analysis was also performed to extract the mean dwell times of the paused and excursion states. These states were categorized according to their FRET efficiency and the states that preceded and followed them. FRET states that were preceded by the baseline and were followed by a higher FRET state were assigned as paused states. FRET states that were preceded by the paused state and were followed by backtracked or baseline states were assigned as excursion states. The dwell times of the states were used to generate dwell time distributions, which were fitted with exponential decay curves. The mean paused state dwell times (τ_{pause}) and the mean excursion dwell times (τ_{excurs}) were extracted from the fits.

Accessible volume (AV) modelling. An *E. coli* RP_O model was used to model the positions of the donor and acceptor dyes during the initial transcription reaction. The model was built using structural alignments to introduce the *E.coli* holoenzyme (PDB 4IGC, Murakami, 2013) and downstream DNA (using the *Tth* RNAP complex with downstream fork-junction DNA; PDB 4G7H, Zhang et al., 2012) in the *Taq* RP_o model (Murakami et al., 2002); downstream DNA beyond position +21 (and up to +35) was further extended as B-DNA. The AV of the donor and acceptor dyes attached to the DNA via a flexible linker were calculated according to (Muschielok et al., 2008). Here, the sterically accessible positions of the dye were obtained from lattice calculations performed on a flexible linker (length=14 Å, width=4.5 Å) attached to the C5 atom of the dT base with the dye modelled as a sphere (with a radius of 7 Å). The accessible volumes were approximated by a 3D Gaussian, the centre of which was approximated as the average position of the dyes. Visualisations and distance measurements between the donor and acceptor average dye positions were performed in PYMOL.

In RP_O, the donor dye was modelled attached to position -15, and the acceptor dye attached to position +20. For each successive nucleotide incorporation step that results in DNA scrunching, we modelled the acceptor dye attached to a position 1-bp upstream of the previous attachment position. Therefore, modelling the acceptor dye attached to +19, +18, +17, +16, and +15 approximated the average positions of the acceptor dye in RP_{ITC3}, RP_{ITC4}, RP_{ITC5}, RP_{ITC6}, and RP_{ITC7}, respectively. The donor dye was modelled still attached to position -15. In all AV models, the donor dye was modelled attached to the non-template strand, and the acceptor dye was modelled attached to the template strand.

REFERENCES

Bronson, J.E., Fei, J., Hofman, J.M., Gonzalez, R.L., and Wiggins, C.H. (2009). Learning rates and states from biophysical time series: a Bayesian approach to model selection and single-molecule FRET data. Biophys. J. *97*, 3196–3205.

Ha, T., and Tinnefeld, P. (2012). Photophysics of fluorescent probes for single-molecule biophysics and super-resolution imaging. Annu. Rev. Phys. Chem. *63*, 595–617.

Morichaud, Z., Chaloin, L., and Brodolin, K. (2016). Regions 1.2 and 3.2 of the RNA Polymerase σ Subunit Promote DNA Melting and Attenuate Action of the Antibiotic Lipiarmycin. J. Mol. Biol. *428*, 463–476.

Murakami, K.S., Masuda, S., Campbell, E.A., Muzzin, O., and Darst, S.A. (2002). Structural basis of transcription initiation: an RNA polymerase holoenzyme-DNA complex. Science *296*, 1285–1290.

Murakami, K.S. (2013) X-ray crystal structure of *Escherichia coli* RNA polymerase σ70 holoenzyme. J.Biol.Chem. *288*, 9126–34.

Muschielok, A., Andrecka, J., Jawhari, A., Brückner, F., Cramer, P., and Michaelis, J. (2008). A nano-positioning system for macromolecular structural analysis. Nat. Methods *5*, 965–971.

Tupin, A., Gualtieri, M., Leonetti, J.-P., and Brodolin, K. (2010). The transcription inhibitor lipiarmycin blocks DNA fitting into the RNA polymerase catalytic site. EMBO J. *29*, 2527–2537.

Uphoff, S., Gryte, K., Evans, G., and Kapanidis, A.N. (2011). Improved temporal resolution and linked hidden Markov modeling for switchable single-molecule FRET. Chemphyschem *12*, 571–579.

Zhang, Y., Feng, Y., Chatterjee, S., Tuske, S., Ho, M.X., Arnold, E., and Ebright, R.H. (2012). Structural basis of transcription initiation. Science *338*, 1076–80.

FIGURE S1 (related to Figure 1)

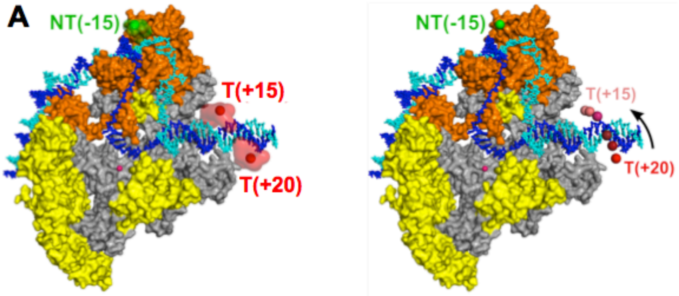

Acceptor position in RP_o	Equivalent RP_{ITC} state	Distance R_{model} (Å)	E_{model}	E_{exp} (E^*)	$E_{exp,\ corr}$	Distance R_{exp} (Å)
+20	ITC2	107	0.04	0.22±0.01	0.04	105.3
+19	ITC3	102	0.05			
+18	ITC4	92	0.09	0.32±0.01	0.13	85.1
+17	ITC5	82	0.16	0.36±0.01	0.21	77.3
+16	ITC6	76	0.23	(0.37±0.01)	(0.22)	76.6
+15	ITC7	73	0.27	(0.45)	(0.31)	70.8

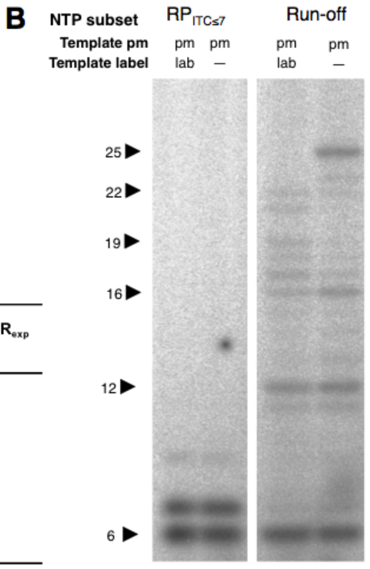

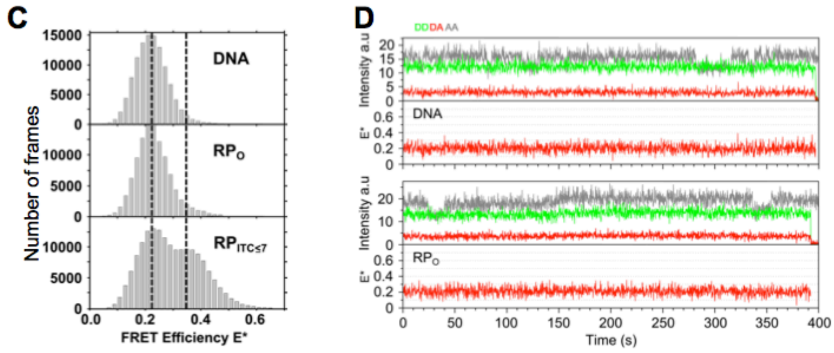

Figure S1. A single-molecule FRET assay for monitoring initial transcription in real-time.

(A) Fluorophore movement during initial transcription. Left, accessible-volume model of the donor fluorophore attached to -15 in RP_O and in RP_{ITC7}, and the acceptor dye attached to +20 in RP_O and +15 in RP_{ITC7}. The accessible volumes and mean positions of the fluorophores are shown. In both positions, the acceptor dye does not clash with protein. Right, accessible-volume model of RNAP during initial transcription. The acceptor dye is attached at position +20 and moves towards the donor during initial transcription. The final position in the model is +15, which corresponds to RP_{ITC7}. The distances between the donor fluorophore at position -15 and the acceptor fluorophore at various positions on the downstream DNA (as determined using accessible-volume modeling) is shown on the Table in the lower panel, along with the expected FRET efficiencies E_{model}. For a comparison, the measured uncorrected FRET efficiencies E_{exp} are shown, along with the corrected FRET efficiencies $E_{exp,\ corr}$ and the corresponding experimental distances R_{exp}.; the first decimal point is shown in order to emphasize the expected *change* in distance at the point of the FRET dynamic range. For the assignment of FRET states to RP_{ITC} complexes, see main text.

(B) *In vitro* transcription as a functional test for the labelled, pre-melted promoter fragment. [α^{32}P]GTP was used in both panels. The initial transcribed sequence is 5'-AAUUGUG-3'. Left panel, testing for any effects of the donor and acceptor fluorophores on the abortive profile of RNAP on the pre-melted lac*CONS* promoter. Right panel, testing for any effects of the donor and acceptor fluorophores on promoter escape and early elongation by RNAP on the pre-melted *lac*CONS promoter.

(C) Immobilized free promoter DNA and RP_{ITC2} show no fluctuations to higher FRET values. Top panel: stacked E* histograms of surface-immobilized DNA (top). Middle panel: RPo plus ApA (RP_{ITC2}). Bottom panel: $RP_{ITC\leq7}$. Each histogram was constructed using data from six fields-of-view. The NTP reaction mixture was added before starting data acquisition. Frame rate is 20 ms.

(D) Example time-traces of DNA (top) and RP_o plus ApA (bottom). FRET remained constant at E*~0.23 for many minutes. ATTO647N intensity state changes can be seen in the AA channel. Frame time: 200 ms.

FIGURE S2 (related to Figure 2)

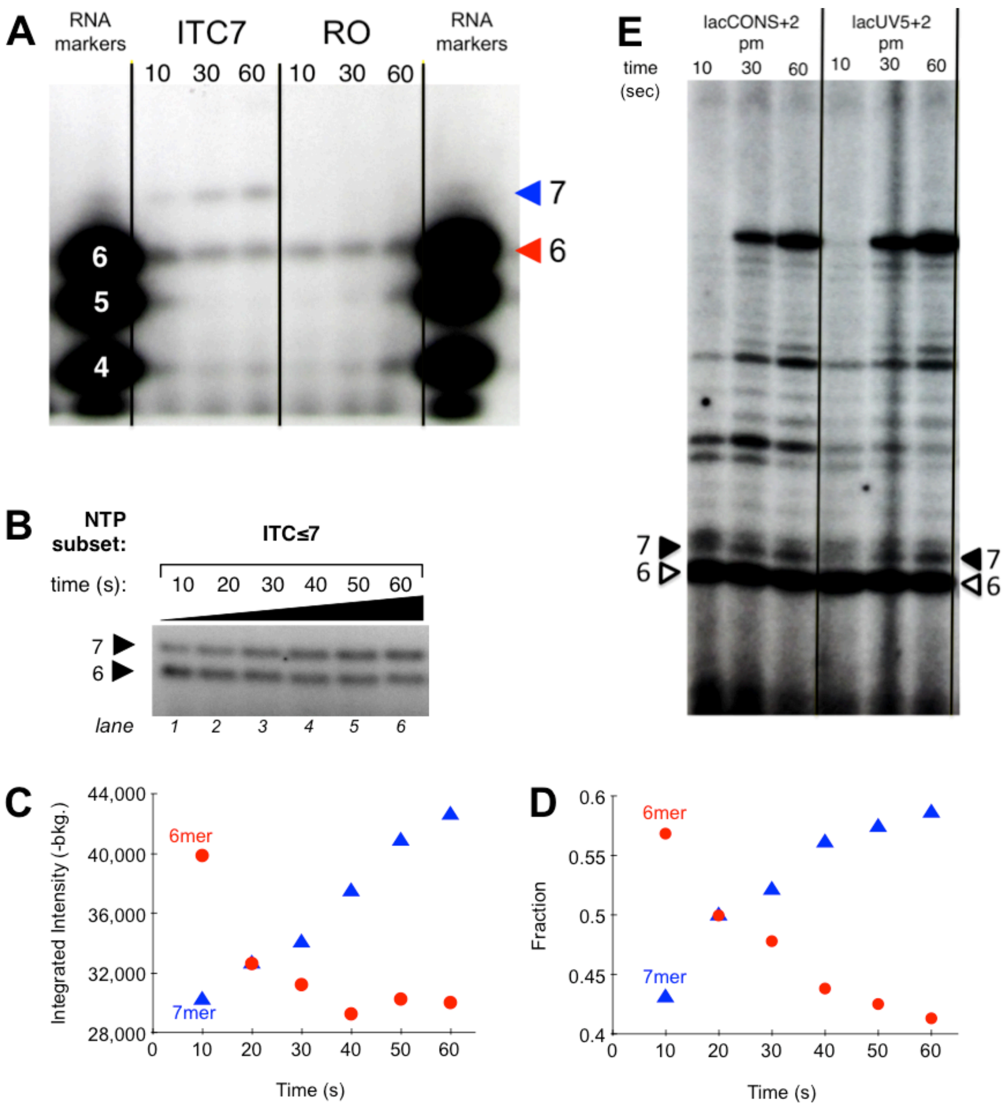

Figure S2. Quantitation of *in vitro* transcription reaction time-points.

(A) Length assignment of short RNA products. *In vitro* transcription gels of RNA products produced by transcription complexes able to synthesize up a 7-nt RNA ("ITC7") and up to a run-off product ("RO"), compared with RNA markers. In both cases, the 5'-end of the RNA is phosphorylated; in the case of transcription, this is achieved by using pApA as a dinucleotide primer. RNA markers are phosphorylated, and carry the exact sequence of the first *lac*CONS (or *lac*UV5) transcripts: the 4-mer is 5'-pAAUU-3', the 5-mer is 5'-pAAUUG-3' and the 6-mer is 5'-pAAUUGU-3'. The mobility comparison clearly shows that the main band in ITC7 as well as the run-off reaction is the 6-mer. Note that although the gel is continuous, black lines have been added for annotation and clarity.

(B) *In vitro* transcription reactions under $RP_{ITC\leq7}$ conditions (for details, see Fig. 2C) reveal a pause event after synthesis of a 6-mer RNA product. Transcription products were detected by phosphorimaging (Fujifilm).

(C-D) RNA-band quantitation using ImageJ (panel C); the amount of the 6- vs. 7-mer product was normalized to the sum of 6- and 7-mer band intensities at each time-point (panel D).

(E) Pausing on *lac*UV5 promoter. *In vitro* transcription gels under transcription runoff conditions (using all NTPs) showing the presence of a 6-mer pause both on *lac*CONS+2 and *lac*UV5 promoters. The promoters differ on the strength of their -35 element (lacCONS+2: TTGACA, matching the -35 consensus; *lac*UV5+2: TTTACA, having a 5 out of 6 match to the consensus) and the degree of match to the consensus length for the -10/-35 spacer (*lac*CONS+2, 17-bp, matching the consensus; *lac*UV5, 18-bp). The profile of short products (up to 10 nt) which reports on pausing after synthesis of a 6-mer, is identical for both promoters. Note that although the gel is continuous, black lines have been added for annotation and clarity.

FIGURE S3 (related to Figure 4)

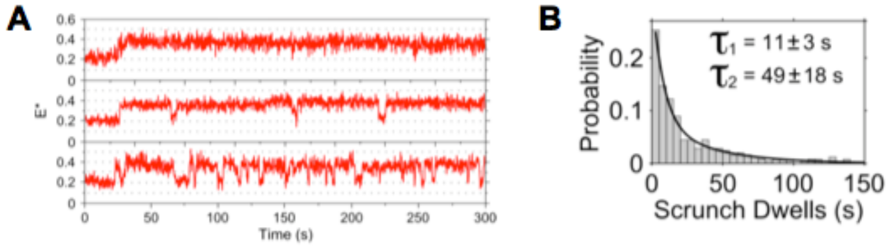

Figure S3. The main initial transcription behaviors in RP$_{ITC\leq7}$ are independent of the bubble mismatch. Frame time: 200 ms.

(A) Example time-traces of stable scrunched (top), and abortive cycling (middle and bottom) immobilized RP$_{ITC\leq7}$ complexes formed using fully complementary DNA (dsDNA).

(B) Dwell-time distribution for dsDNA using abortive cycling data (*N*=250). The scrunched state lifetimes result from the fit of the distribution to a bi-exponential decay; the amplitudes of the short and long lifetimes are ~70% and ~30%, respectively.

FIGURE S4 (related to Figure 6)

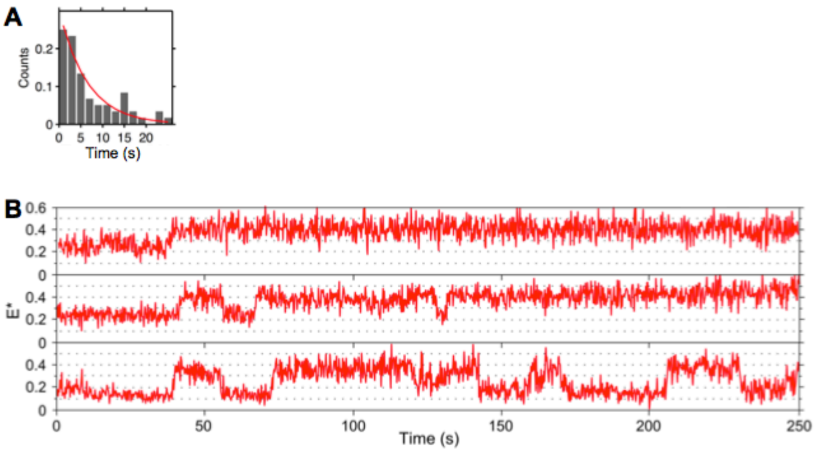

Figure S4. Analysis of complexes provided with all four NTPs to allow promoter escape.

(A) Dwell-time histogram for the escape pause; the average dwell time is 7.7 s (*N*=62).

(B) Example time-traces of transcription complexes that, despite being provided the full set of NTPs for promoter escape, show abortive-cycling behaviors similar to those seen for $RP_{ITC\leq7}$ complexes.
